# Dysregulated Ribosome Biogenesis Is a Targetable Vulnerability in Triple-Negative Breast Cancer: MRPS27 as a Key Mediator of the Stemness-inhibitory Effect of Lovastatin

**DOI:** 10.7150/ijbs.94058

**Published:** 2024-03-18

**Authors:** Chanjuan Zheng, Hui Yao, Lu Lu, Hongqi Li, Lei Zhou, Xueyan He, Xi Xu, Hongzhuo Xia, Siyu Ding, Yiyuan Yang, Xinyu Wang, Muyao Wu, Lian Xue, Sisi Chen, Xiaojun Peng, Zhongyi Cheng, Yian Wang, Guangchun He, Shujun Fu, Evan T. Keller, Suling Liu, Yi-zhou Jiang, Xiyun Deng

**Affiliations:** 1 Key Laboratory of Model Animals and Stem Cell Biology in Hunan Province, Department of Pathophysiology, Hunan Normal University School of Medicine, Changsha, Hunan, China.; 2 Key Laboratory of Translational Cancer Stem Cell Research, Hunan Normal University, Changsha, Hunan, China.; 3 Fudan University Shanghai Cancer Center & Institutes of Biomedical Sciences, State Key Laboratory of Genetic Engineering, Fudan University, Shanghai, China.; 4 Jingjie PTM BioLab Co. Ltd., Hangzhou Economic and Technological Development Area, Hangzhou, China.; 5 Department of Urology and Biointerfaces Institute, University of Michigan, Ann Arbor, Michigan, USA.; 6 Key Laboratory of Breast Cancer in Shanghai, Department of Breast Surgery, Precision Cancer Medicine Center, Fudan University Shanghai Cancer Center, Shanghai, China.; 7 Department of Oncology, Shanghai Medical College, Fudan University, Shanghai, China.

**Keywords:** Triple-negative breast cancer, Ribosome biogenesis, Lovastatin, Stemness, MRPS27

## Abstract

Triple-negative breast cancer (TNBC) is the most aggressive subtype of breast cancer with limited effective therapeutic options readily available. We have previously demonstrated that lovastatin, an FDA-approved lipid-lowering drug, selectively inhibits the stemness properties of TNBC. However, the intracellular targets of lovastatin in TNBC remain largely unknown. Here, we unexpectedly uncovered ribosome biogenesis as the predominant pathway targeted by lovastatin in TNBC. Lovastatin induced the translocation of ribosome biogenesis-related proteins including nucleophosmin (NPM), nucleolar and coiled-body phosphoprotein 1 (NOLC1), and the ribosomal protein RPL3. Lovastatin also suppressed the transcript levels of rRNAs and increased the nuclear protein level and transcriptional activity of p53, a master mediator of nucleolar stress. A prognostic model generated from 10 ribosome biogenesis-related genes showed outstanding performance in predicting the survival of TNBC patients. Mitochondrial ribosomal protein S27 (MRPS27), the top-ranked risky model gene, was highly expressed and correlated with tumor stage and lymph node involvement in TNBC. Mechanistically, MRPS27 knockdown inhibited the stemness properties and the malignant phenotypes of TNBC. Overexpression of MRPS27 attenuated the stemness-inhibitory effect of lovastatin in TNBC cells. Our findings reveal that dysregulated ribosome biogenesis is a targetable vulnerability and targeting MRPS27 could be a novel therapeutic strategy for TNBC patients.

## Introduction

Triple-negative breast cancer (TNBC), which is characterized by lack of estrogen receptor (ER) and progesterone receptor (PR) expression and lack of overexpression/amplification of human epidermal growth factor receptor 2 (HER2), is the most aggressive subtype of breast cancer with the least favorable outcomes. Patients with metastatic TNBC rapidly develop resistance to chemotherapy, resulting in a median overall survival (OS) of 8-13 months 1, 2. Despite the advances in breast cancer treatment, the prognosis for TNBC patients has seen little improvement over the past few decades 3. Novel therapeutic strategies, including poly (ADP-ribose) polymerase (PARP) inhibition, immunotherapy, and inhibition of signaling pathways such as the androgen receptor, PI3K/AKT/mTOR, Notch, and EGFR pathways, are being actively explored 4. Indeed, PARP inhibitors such as olaparib and veliparib 5, immune checkpoint inhibitors such as pembrolizumab 6, 7, and antibody-drug conjugates (ADCs) such as sacituzumab govitecan 8 have demonstrated activity in a subset of TNBC patients. However, the overall benefit of these novel therapeutics for the majority of TNBC patients is limited 9. Developing and understanding of the characteristic changes that occur intracellularly within tumor cells can be used to identify potential therapeutic targets in TNBC.

Ribosome biogenesis, which contributes to the synthesis of proteins required for the cell's diverse functions and consumes up to 80% of a cell's energy, represents the most metabolically vulnerable process of the cell 10, 11. Ribosome biogenesis occurs primarily in the nucleolus and involves RNA Pol I-directed transcription of rRNAs from ribosomal DNA and assembly of rRNAs with ribosomal proteins to form the mature 80S cytosolic ribosome. The nucleolus is a membraneless organelle arranged into a tripartite structure consisting of a fibrillar center (FC), a dense fibrillar component (DFC), and a granular component (GC). These cellular divisions are responsible for rRNA transcription, rRNA processing, and ribosome assembly, respectively 12, 13. Cancer cells often show increased ribosome biogenesis to cope with a rise in protein synthesis and sustain unrestricted tumor growth and spread 14, 15. Emerging evidence suggests that cancer cells harbor aberrant ribosomes (onco-ribosomes) and/or extra-ribosome ribosomal proteins that facilitate oncogenic transformation, promote metabolic reprogramming, and confer therapeutic resistance 11. Ribosome biogenesis stress, i.e., nucleolar stress, is a series of cellular abnormalities related to the structural and functional aspects of the nucleolus induced by diverse cellular insults, leading to dysfunction of protein translation and deleterious cellular consequences including cell death 16, 17. Thus, targeting the ribosome biogenesis pathway to induce nucleolar stress represents an innovative therapeutic strategy that holds great promise for cancer therapy 10, 18.

Statins, inhibitors of 3-hydroxy-3-methylglutaryl coenzyme A (HMG-CoA) reductase, have been used as cholesterol-lowering drugs for over four decades with a generally acceptable toxicity profile 19. Recently, there has been tremendous interest in repurposing these safe, well-tolerated, and inexpensive statins as anti-cancer agents 20, 21. Observational cross-sectional clinical studies have demonstrated that the use of statins, particularly lipophilic statins, is associated with decreased risk of breast cancer recurrence 22 or decreased mortality due to breast cancer and all causes 23. Furthermore, interventional studies revealed that use of statins may improve the efficacy of neoadjuvant chemotherapy in locally advanced breast cancer patients 24. A recently completed clinical trial (NCT03454529) showed that simvastatin reduced the mortality of breast cancer patients without causing serious adverse events. Studies from our laboratory 25, 26 and others 27, 28 have demonstrated that lovastatin, the first FDA-approved naturally derived lipid-lowering drug 29, preferentially inhibits TNBC cells in preclinical models. Several mechanisms have been proposed for statin's anticancer activity including inducing programmed cell death and targeting the tumor microenvironment 21, 29. However, the role of the ribosome biogenesis pathway in lovastatin's action in TNBC remains unknown.

In this study, we demonstrate that lovastatin inhibits TNBC cancer stem cells (CSCs) *in vitro* and *in vivo*. Through isobaric tags for relative and absolute quantitation (iTRAQ)-based proteomics profiling of TNBC patient tissues and bioinformatics analysis of TCGA-BRCA RNA-seq data, we found that dysregulation of ribosome biogenesis is a characteristic feature in TNBC. Furthermore, we investigated the role of the ribosome biogenesis pathway as a therapeutic vulnerability in TNBC CSCs and constructed a prognostic model based on 10 ribosome biogenesis-related genes in TNBC patients. We also found MRPS27 (mitochondrial ribosomal protein S27) was important for the stemness properties of TNBC and could mediate the stemness-inhibitory effect of lovastatin in TNBC cells. Our findings reveal that dysregulated ribosome biogenesis is a targetable vulnerability in TNBC and targeting MRPS27 could be a novel therapeutic strategy for TNBC patients.

## Materials and Methods

### Cell culture

TNBC (MDA-MB-231, MDA-MB-468, MX-1, BT549, HCC1806, 4T1, and HCC1937) and non-TNBC (MCF-7, T47D, MDA-MB-453, BT474, ZR-75-1) cell lines were obtained from the CAS Shanghai Cell Resources Center and cultured according to the guidelines of the provider. None of these cell lines was listed in the ICLAC database of commonly cross-contaminated or misidentified cell lines (http://iclac.org/databases/cross-contaminations/).

### Enrichment and characterization of breast cancer stem cells

The CSCs were enriched from breast cancer cells based on the CD44^+^/CD24^-^ phenotype and the sphere-forming ability 30, 31. CD44^+^/CD24^-^ cells were enriched using the human CD44^+^/CD24^-^ breast cancer stem cell isolation kit (Cat# MAGH111, R&D Systems, Minneapolis, MN, USA) and maintained in ultra-low attachment 6-well plates (2,500 - 5,000 cells/well) in serum-free stem cell medium to obtain sphere-forming cells (SFCs). The culture medium consisted of DMEM/F12 (Life Technologies, Grand Island, NY, USA) supplemented with 1× B27 (Life Technologies), 20 ng/mL EGF (Prospec, East Brunswick, NJ, USA), 20 ng/mL bFGF (Prospec), 0.4% BSA (Sigma-Aldrich, St Louis, MO, USA), and 4 µg/mL Insulin (Genview, Pompano Beach, FL, USA). The CSC phenotype of the SFCs was verified in nude mice (data not shown).

### Sphere-forming and self-renewal assays

The sphere-forming or self-renewal capacity of breast cancer cells was measured by plating the parental cells or SFCs in serum-free stem cell culture medium and calculating the areas of tumorspheres formed 5 days after treatment as previously described 32.

### Cell viability assay

An Alamar blue fluorescent assay was used to determine cell viability. Briefly, the cells were seeded in 96-well plates at 1 × 10^4^ cells/well in 100 μl volume. The next day, the cells were treated with different concentrations of lovastatin in 6 replicates for 48 - 72 h. Prior to harvest, 20 μl of CellTiter blue cell viability assay reagent (Cat# G7570, Promega, Madison, WI, USA) was added to each well and the culture continued for 1 - 4 h. Fluorescence signal (530/590 nm) was measured using a Synergy 2 Microplate Reader (BioTek, Waltham, MA, USA). IC_50_ values were calculated using the GraphPad Prism 5 software.

### Key reagents

Lovastatin (Mevinolin) was purchased from Selleck (Cat# S2061, Houston, TX, USA) or Abcam (Cat# ab120614, Cambridge, MA, USA) and dissolved in DMSO at a stock concentration of 30 mM and stored at -80°C before use. The information of primary antibodies used in this study is available in **Supplementary Table S1**.

### Mouse model of orthotopic tumor growth

Female nude mice aged 4 - 5 weeks were purchased from Hunan SJA Laboratory Animals Co. Ltd. and maintained in sterile cages in a pathogen-free aseptic environment with the 12h/12h light/dark cycle. The SFCs (2 × 10^3^/50 μL/mouse) or transfected parental cells (1 × 10^6^/50 μL/mouse) resuspended in cold 1× PBS were mixed with growth factor-reduced Matrigel (BD Biosciences, Franklin Lakes, NJ, USA) at a 1:1 ratio and injected into the 4^th^ mammary fat pad of the mice. Two weeks after cell inoculation when the tumors reached 5 - 8 mm in diameter, the mice were randomly grouped based on tumor sizes. Lovastatin (2 mg/kg b.w.) or vehicle (saline) was administered twice weekly through intragastric gavage for three weeks. Tumor growth was monitored by measuring the major (*a*) and minor (*b*) axes of the tumor using a caliper twice weekly. The tumor volume (*V*) was estimated using the equation *V* = (*a* × *b*^2^)/2. At the end of drug treatment, the mice were sacrificed, and the tumors resected, weighed, and photographed. A portion of the tumor tissue was fixed in 4% buffered formaldehyde and subjected to routine paraffin-embedding, microtome sectioning, followed by H&E staining or immunohistochemistry.

The protocols for the mouse model of orthotopic tumor growth were approved by the Hunan Normal University Institutional Animal Care and Ethics Committee and carried out in accordance with the guidelines stated in the International Guiding Principles for Biomedical Research Involving Animals.

### Tumorigenesis of patient-derived xenografts (PDXs)

The tumorigenesis of TNBC PDX model was performed according to our published procedure 33. Briefly, female nude mice (3 - 4 weeks old) obtained from Charles River Laboratories (Beijing, China) were housed under specific pathogen-free conditions in the Department of Laboratory Animal Science of Fudan University Shanghai Cancer Center. All mouse experiments were conducted in accordance with standard operating procedures as recommended in the Guide for the Care and Use of Laboratory Animals of Fudan University. The Fudan University Shanghai Cancer Center Institutional Review Board approved the mouse experiment protocol (JS-082) and the use of human samples (050432-4-1212B) for this study. Different numbers (50,000, 5,000, or 500) of cancer cells from the TNBC PDXs (FD-11, invasive ductal carcinoma, age 55, ER (-), PR (-), HER2 (2+), FISH (-), CK5/6 (+), Ki67 (+>80%)) were injected into the 4^th^ mammary fat pad on both sides of each mouse (*n* = 2 tumors/mouse × 5 mice/group). The mice were treated with lovastatin (2 mg/kg or 10 mg/kg b.w.) or saline, monitored for tumor growth, and sacrificed, and stem cell frequency calculated.

### ALDEFLUOR assay

Tumor cells from the PDX tumors were dissociated into single cells, resuspended in assay buffer containing ALDEFLUOR substrate (StemCell, Cambridge, USA), and analyzed and quantified according to our published methods 34.

### LC-MS/MS analyses of global proteomics and lysine acylation profiling

The SFCs were treated with lovastatin (1 μM) or vehicle in stem cell medium at 37°C for 48 h. The cells were then collected by centrifugation and snap-frozen in liquid nitrogen, followed by protein extraction and trypsin digestion. The resulting peptides were labeled with tandem mass tag (TMT) isobaric reagents and fractionated by strong cation exchange chromatography. Lysine-acylated peptides were immunoprecipitated with each type of pan-lysine acylation antibody-conjugated beads. Enriched peptides were analyzed by liquid chromatography coupled to an Orbitrap Q Exactive^TM^ Plus or Q Exactive^TM^. Non-enriched peptides (for global proteomics) were fractionated by reverse-phase HPLC using the Agilent 300 Extend C18 column followed by LC-MS/MS analysis. The resulting MS/MS data was processed using MaxQuant with integrated Andromeda search engine (v.1.5.2.8). Tandem mass spectra were searched against *Swiss-Prot human* database concatenated with reverse decoy database. False discovery rate (FDR) thresholds for proteins, peptides, and modification sites were specified at 1%. Minimum peptide length was set at 7. For quantification method, TMT 6-plex was selected. The site localization probability was set at ≥ 0.75. The relative changes of lysine-acylated proteins were normalized to the respective protein level revealed by global proteomic profiling.

### Functional enrichment analysis

Gene Ontology (GO) analysis, Kyoto Encyclopedia of Genes and Genomes (KEGG) pathway enrichment analysis, and Gene Set Enrichment Analysis (GSEA) were performed using the R/Bioconductor package “*clusterProfiler*”. For enriched gene sets, a normalized *P* < 0.05 and a false discovery rate (FDR) q < 0.25 were considered statistically significant. The protein-protein interaction (PPI) network was generated using the STRING (https://string-db.org/) database.

### Immunofluorescence - laser scanning confocal microscopy

The cells were grown on coverslips and treated with lovastatin, fixed with 4% paraformaldehyde, and subjected to indirect immunofluorescence. The cells were imaged using a Leica TCS SP8 Confocal Microscope (Wetzlar, Germany). Bidirectional scanning and 4× line averaging were used on 2,048 × 2,048 resolution with zoom adjusted according to the field of interest. The information of primary antibodies used for immunofluorescence is available in **Supplementary Table S1**.

### Cell lysate preparation and western blot analysis

Whole cell lysates or cytoplasmic/nuclear fractions were prepared and quantified according to the standard procedure. Western blot analysis was performed by separating the cell lysates on 10% or 12% SDS-PAGE gels, followed by electroblotting, primary and secondary antibody incubation, and ECL development. When necessary, band intensities were quantified using the Bio-Rad Image Lab software and normalized to the house-keeping protein. The information of primary antibodies for western blots is also available in **Supplementary Table S1**.

### qRT-PCR

Total RNA was extracted from the cells with the RNA Isolator reagent (Vazyme, Nanjing, China). The purified RNA was reverse-transcribed to cDNA using HiScript 1st Strand cDNA Synthesis Kit (Vazyme). Quantitative PCR reactions following the run conditions described in the reference for each primer pair were conducted using the SuperMix qPCR reagent (Vazyme) in a CFX Connect Real-Time PCR Detection System (Bio-Rad, Hercules, CA, USA). The information of PCR primers used in this study is available in **Supplementary Table S2**.

### Human breast cancer tissues and iTRAQ proteomics profiling

Breast cancer tissues were collected from 40 patients (10 TNBC and 30 non-TNBC) who were hospitalized and received surgery in Hunan Cancer Hospital (Changsha, China) without pre-operative radiotherapy or chemotherapy. All patients were diagnosed with invasive ductal breast cancer by two senior pathologists. Molecular subtypes were routinely classified according to immunohistochemical staining of ER, PR, and HER2 as well as *in situ* hybridization of HER2/ErbB2. Each tissue sample was divided into two parts, one for histopathological examination and the other for proteomics analysis. The study was approved by the Ethics Committee of Hunan Cancer Hospital and performed in compliance with all relevant ethical regulations regarding research involving human participants. The patient characteristics are summarized in** Supplementary Table S3**. Proteomics profiling of patient tissue samples was performed as described in our previous publication 35.

### TCGA-BRCA RNA-seq and clinical data

The TCGA RNA-seq and clinical data of breast cancer patients were downloaded from Genomic Data Commons (GDC) Data Portal (https://portal.gdc.cancer.gov/) (Data Release 31.0) using the R software (version 4.0.3). This study included a total of 1,102 cases of breast cancer samples (231 TNBC and 871 non-TNBC cases) from the TCGA-BRCA database. We employed the PAM50 subtyping classifier to identify the basal-like (a surrogate of TNBC) and other breast cancer subtypes 36.

### Validation datasets

The Gene Expression Omnibus (GEO) validation cohort (GSE58812), which included 107 TNBC patients, was downloaded from the GEO database. Another validation cohort (Fudan University Shanghai Cancer Center, FUSCC), which included 465 TNBC patients, was obtained from the National Omics Data Encyclopedia (NODE) by pasting the accession (OEP000155).

### Construction, evaluation, and validation of a prognostic model

Ribosome biogenesis-related genes were collected from the Molecular Signatures Database (MSigDB) (https://www.gsea-msigdb.org/gsea/msigdb) 37 by searching the keywords “ribosome biogenesis” or “ribosome”. The differentially expressed genes were analyzed using the R/Bioconductor package “*limma*”. Univariate Cox regression, least absolute shrinkage and selection operator (LASSO) Cox, and multivariate Cox regression analyses were used to construct a prognostic model based on ribosome biogenesis-related genes followed by calculation of the risk score according to the following formula.

Risk score = 
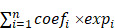


TNBC patients were categorized into the high- and low-risk groups based on the median value. Kaplan-Meier analysis of patient survival was performed using the R packages “*survival*” and “*survminer*”. Time-dependent receiver-operating characteristic (ROC) curves were analyzed by comparing the area under the curve (AUC) values using the R package “*survivalROC*”. The validation sets (GSE58812 and FUSCC) of TNBC patients were used to further validate the predictive performance of the model.

### Immunohistochemistry

Immunohistochemical staining was carried out using the PV-9000 plus poly-HRP anti-mouse/rabbit IgG detection system as described in our previous study 26. The details of primary antibodies used for immunohistochemistry are described in **Supplementary Table S1**. For quantification, the tissue sections were scanned using Automated Quantitative Pathology Imaging System (Vectra, PerkinElmer, Hopkinton, MA, USA) and the immunoreactivity score was calculated as described.

### Ribosome profiling

Ribosome profiling (Ribo-seq) was performed to detect the difference in translation efficiency as measured by the ratio of ribosome-protected mRNA fragments (ribosome footprints) to unprotected mRNA fragments as described 38. Briefly, MDA-MB-231 cells transfected with shMRPS27 or control shRNA pretreated with cycloheximide (CHX, 100 µg/ml, 2 min) were lysed, nuclease-digested to generate ribosome footprints, processed to remove unprotected mRNA and rRNA. Ribosome footprints were purified followed by library construction, deep sequencing using the Illumina HiSeq^TM^ X10 platform, and data analysis. Elsevier Pathway Collection was performed to reveal the pathways affected by MRPS27. Ribo-seq and data analyses were performed by Gene Denovo Biotechnology Co. Ltd (Guangzhou, China).

### Knockdown and overexpression of MRPS27

The short hairpin RNAs (shRNAs) for MRPS27 were designed and constructed by GenePharma Corporation (Shanghai, China), and were utilized to silence expression of MRPS27 in TNBC cell lines (MDA-MB-231 and BT549). The specific silencing was confirmed by western blot analysis and qRT-PCR. The sequences of shRNAs for knockdown of MRPS27 are available in **Supplementary Table S4**. MRPS27 overexpression plasmid was designed and constructed by Genecreate Corporation (Wuhan, China) by inserting MRPS27 cDNA into the pcDNA3.1-3xFlag vector. The MRPS27-overexpressing plasmid or the vector was transiently transfected into TNBC HCC1937 cells using Lipofectamine 8000. The overexpression efficiency for MRPS27 was confirmed by western blot analysis.

### Transwell migration and invasion assays

Cell migration and invasion assays were conducted with PET membranes (8 μm pore size, 24-well plate) coated without (for migration) or with (for invasion) Matrigel (Corning, Tewksbyry, MA, USA). Single cell suspension in serum-free medium was added to the upper wells (20,000 cells/well), while migration-inducing medium (with 10% FBS) was added to the lower wells. After 24 h, the cells on the top surface of the chambers were scraped off with a cotton swab. The cells on the lower surface of the membranes were fixed with methanol and stained with 0.1% crystal violet solution. The cells that penetrated the membrane were evaluated microscopically and quantified from three random fields per membrane.

### Colony formation assay

For the colony formation assay, the cells (in single-cell suspension) were seeded in 6-well plates (1,000 cells/plate). The media were refreshed every 3 days. After 14 days of incubation, the media were discarded and the colonies were washed with PBS, and then stained with 0.1% crystal violet solution for 15 min. The colonies were then photographed followed by counting and quantification.

### GEPIA2 analysis

The correlation of MRPS27 expression with the nucleolar stress-related proteins and stemness-related proteins in breast cancer was analyzed using the online database Gene Expression Profiling Interactive Analysis 2 (GEPIA2) (http://gepia2.cancer-pku.cn/#index). The Spearman method was used to determine the correlation coefficient.

### Statistical analyses

The quantitative data were expressed as mean ± SEM and were analyzed using the *t* test. The qualitative data were analyzed using the chi-square test. All statistical analyses were performed with the R software (version 4.0.3). *P* values < 0.05 were considered statistically significant.

## Results

### Lovastatin inhibits TNBC stemness* in vitro* and *in vivo*

**Figure 1:**
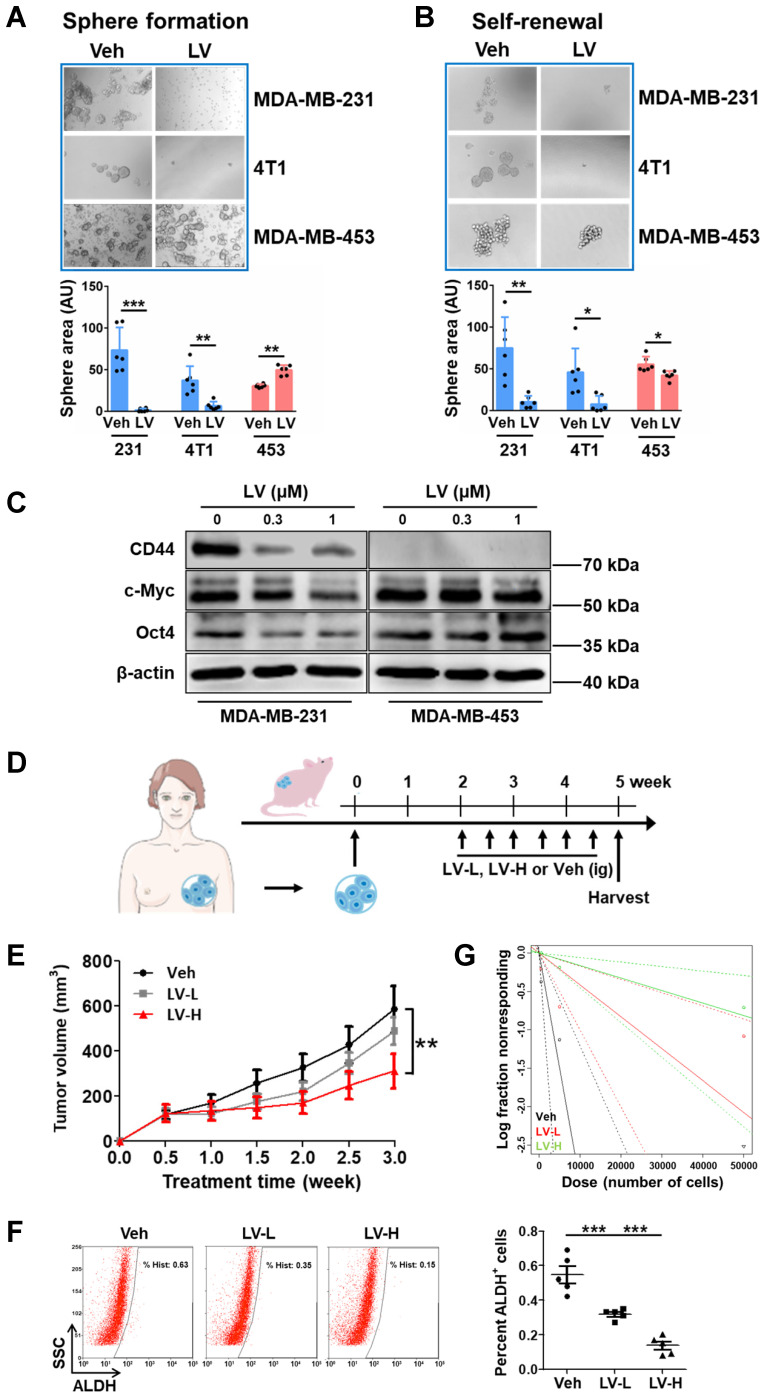
** Lovastatin inhibits TNBC cancer stem cells *in vitro* and in a PDX model.** (**A**) Sphere formation after treatment of the parental cells for 5 d with vehicle or lovastatin (1 μM). Bottom, quantifications of the areas of tumorspheres in TNBC (blue) and non-TNBC (red) cells. (**B**) Self-renewal after treatment of sphere-forming cells (SFCs) for 5 d with vehicle or lovastatin (0.3 μM). (**C**) Western blot analysis for stemness-related proteins after treatment with different concentrations of lovastatin for 48 h. (**D**) Schematic of the PDX model. (**E**) Growth curves of TNBC PDX tumors after lovastatin or vehicle treatment. *n* = 2 tumors/mouse × 5 mice per group. (**F**) ALDEFLUOR determination of the ALDH^+^ proportion in TNBC PDXs. Right, quantifications of the ALDH^+^ cells (%). (**G**) Stem cell frequency in TNBC PDXs calculated by limiting dilution assay. *P < 0.05, **P < 0.01, ***P < 0.001; Veh, vehicle; LV, lovastatin; LV-L, lovastatin-low dose (2 mg/kg); LV-H, lovastatin-high dose (10 mg/kg); AU, arbitrary unit; PDX, patient-derived xenograft; ig: intragastric administration.

Recently, we reported that lovastatin preferentially inhibited TNBC compared with non-TNBC cells 25, 26. The preferential inhibitory effect of lovastatin on TNBC cells was confirmed using cell viability assay (**Supplementary Fig. S1A**) and microscopic examination (**Supplementary Fig. S1B**). Furthermore, lovastatin inhibited stemness properties including sphere formation (**Fig. 1A**) and self-renewal capacity (**Fig. 1B**) in TNBC MDA-MB-231 and 4T1 cells but not in non-TNBC MDA-MB-453 cells. Finally, lovastatin decreased the protein levels of stemness markers CD44, c-Myc, and Oct4 in MDA-MB-231 cells as compared to no or limited effect on MDA-MB-453 cells (**Fig. 1C**).

We then investigated the anti-CSC potential of lovastatin *in vivo*. For this purpose, a mouse model of orthotopic tumor growth originating from SFCs was used (**Supplementary Fig. S2A**). Mouse weight analyzed before and at the end of the treatment was not significantly altered between the lovastatin- and vehicle-treated groups (data not shown), suggesting mice were able to tolerate lovastatin administration. Lovastatin inhibited the tumor growth in mice inoculated with MDA-MB-231 SFCs but not MDA-MB-453 SFCs (**Supplementary Fig. S2B and C**). We further examined the effect of lovastatin on TNBC patient-derived xenografts (PDXs) by treating the PDX-bearing mice with lovastatin at 2 mg/kg (low dose) or 10 mg/kg (high dose) or vehicle in a similar way as for the mouse model of orthotopic tumor growth (**Fig. 1D**). As expected, lovastatin significantly inhibited PDX tumor growth at a dose of 10 mg/kg (*P* < 0.01), while the lower dose of lovastatin (2 mg/kg) exhibited minimal effect (**Fig. 1E**). Expression of aldehyde dehydrogenase (ALDH), a marker of breast cancer CSCs 39, was decreased by lovastatin in a dose-dependent manner (**Fig. 1F**). Moreover, limiting dilution assay showed that the stem cell frequency was significantly decreased in lovastatin-treated groups compared with the control group (**Fig. 1G and Supplementary Table S5**). Taken together, the data from the *in vitro* and *in vivo* models suggest that lovastatin inhibits the stemness properties of TNBC CSCs.

### Lovastatin targets the ribosome biogenesis pathway and induces nucleolar stress in TNBC cancer stem cells

**Figure 2:**
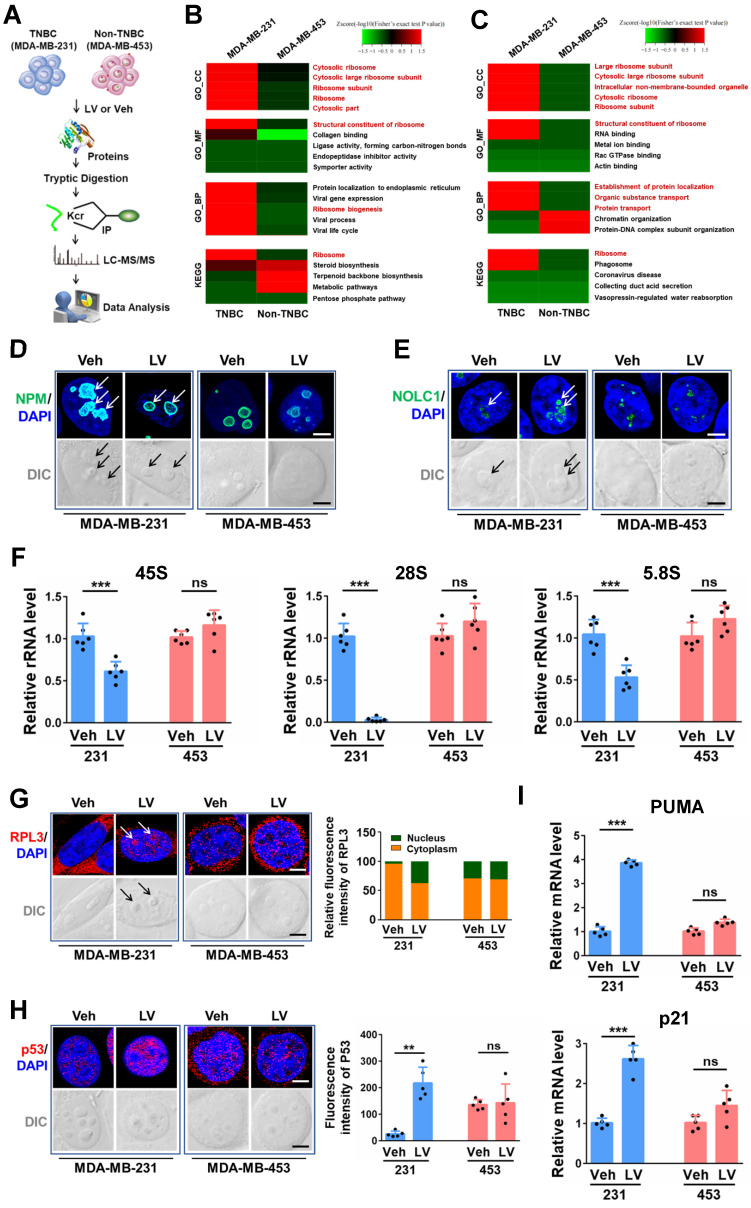
** Lovastatin targets the ribosome biogenesis pathway and induces nucleolar stress in TNBC cancer stem cells.** (**A**) The flow chart for proteomics and Kcr profiling. (**B**) GO and KEGG analyses showing the enrichment of differentially regulated proteins in lovastatin-treated MDA-MB-231 SFCs compared with MDA-MB-453 SFCs based on proteomics profiling. The top 5 enriched pathways are displayed. (**C**) GO and KEGG analyses showing the enrichment of differentially Kcr-modified proteins in lovastatin-treated MDA-MB-231 SFCs compared with MDA-MB-453 SFCs based on Kcr profiling. The top 5 enriched pathways are displayed. (**D and E**) Representative confocal images of immunofluorescence staining for NPM (**D**) or NOLC1 (**E**) in MDA-MB-231 and MDA-MB-453 SFCs after treatment for 48 h with lovastatin (0.3 μM) or vehicle. DIC images reveal the nucleoli (black arrows) as phase-dense structures. NPM and NOLC1 (white arrows) that locate to the nucleolus are indicated. Scale bar = 5 μm. (**F**) Levels of the precursor 45S rRNA and the mature 28S and 5.8S rRNAs in MDA-MB-231 and MDA-MB-453 SFCs treated for 48 h with lovastatin (0.3 μM) or vehicle. (**G** and** H**) Representative confocal images of immunofluorescence staining for RPL3 (**G**) and p53 (**H**). Right, quantifications of fluorescence intensity of RPL3 (nuclear *vs* cytoplasmic) and p53, respectively. (**I**) Expression levels of p53 target genes in SFCs treated for 48 h with lovastatin (0.3 μM) or vehicle. **P < 0.01, ***P < 0.001; Kcr, lysine crotonylation; LV, lovastatin; Veh, vehicle; BP, biological process; MF, molecular function; CC, cellular component; DIC, differential interference contrast; ns, not significant.

Next, we wanted to identify the pathway(s) through which lovastatin exerts its TNBC-inhibitory effects. For this purpose, we performed proteomics profiling of sphere-forming cells (SFCs) derived from MDA-MB-231 *vs* MDA-MB-453 (**Fig. 2A**). GO and KEGG analyses revealed that the main enriched pathways of lovastatin-dysregulated proteins in MDA-MB-231 SFCs were related to ribosome biogenesis, including cytosolic ribosome, ribosome subunit, structural constituent of ribosome, ribosome biogenesis, etc (**Fig. 2B**). We then generated a PPI network based on the STRING platform, which revealed the most prominent cluster of differentially regulated proteins to be the ribosome biogenesis pathway (**Supplementary Fig. S3A**).

Lysine is the most significantly enriched amino acid residue that is found in the nucleolus compared with the cytosol and the nucleus 40. Acylations on lysine residues have been shown to play a critical role in drug-induced cytotoxicity 41. We screened the four most common types of lysine acylations, i.e., acetylation (Kac), malonylation (Kmal), succinylation (Ksucc), and crotonylation (Kcr). While having neglectable effect on Kac and Kmal, lovastatin treatment caused significant changes of Ksucc and Kcr in MDA-MB-231 SFCs but not in MDA-MB-453 SFCs (data not shown). We next performed tandem mass tag (TMT) labeling and affinity enrichment followed by LC-MS/MS to uncover the changes of Ksucc and Kcr modifications and the specific sites. While lovastatin-induced Ksucc modifications in MDA-MB-231 SFCs occurred mainly on proteins involved in cytoskeleton organization 26, Kcr-modified proteins in these cells were mostly enriched in ribosome biogenesis-related categories (**Fig. 2C**). The PPI network again confirmed the clustering of lovastatin-induced Kcr modifications on proteins involved in ribosome biogenesi**s (Supplementary Fig. S3B**). Thus, both proteomics and lysine crotonylation profiling results demonstrate that the ribosome biogenesis pathway is the predominant cellular target of lovastatin in TNBC CSCs.

Subsequently, we determined whether lovastatin could target the ribosome biogenesis pathway to induce nucleolar stress (**Supplementary Fig. S4**). Translocation of nucleophosmin (NPM, also known as B23), the most abundant nucleolar protein in the GC 12, and nucleolar and coiled-body phosphoprotein 1 (NOLC1, also known as Nopp140), a master regulator of the nucleolus organization and rRNA synthesis in the DFC 42, are typical hallmarks of nucleolar stress. In contrast to the more diffuse distribution in the nucleolus of the control cells, NPM in lovastatin-treated MDA-MB-231 SFCs exhibited a perinucleolar pattern (**Fig. 2D**). Concerning NOLC1, we found that lovastatin increased its protein level as well as promoted the formation of its ring structure in the nucleolus in MDA-MB-231 but not MDA-MB-453 SFCs (**Fig. 2E**). Increased protein level of NOLC1 in the nucleus of MDA-MB-231 SFCs was confirmed using western blot analysis (**Supplementary Fig. S5**).

We further tested whether lovastatin treatment affects the transcript levels of rRNAs. As expected, qRT-PCR analysis revealed that the levels of the precursor 45S rRNA and the mature 28S and 5.8S rRNAs were suppressed by lovastatin in MDA-MB-231 but not MDA-MB-453 SFCs (**Fig. 2F**). We next examined whether lovastatin induces translocation of ribosomal proteins in TNBC CSCs. We focused on RPL3 and RPS10, which were shown to be dysregulated by lovastatin based on Kcr profiling results (**Supplementary Fig. S3B**). We found that lovastatin treatment resulted in apparent decrease of RPL3 in the cytoplasm and its concomitant accumulation in the nucleus, particularly in the nucleolus, in MDA-MB-231 but not MDA-MB-453 SFCs (**Fig. 2G**). We also observed an increase in the protein level of RPS10 in the nucleus following treatment with lovastatin in MDA-MB-231 SFCs (data not shown).

Nucleolar stress occurs predominantly through the p53-dependent pathway in response to cancer therapeutic drugs 43. We found that there was an increase in the protein level of p53 in the nucleus of lovastatin-treated MDA-MB-231 but not MDA-MB-453 SFCs (**Fig. 2H**). Correspondingly, increased transcriptional activity of p53 was demonstrated by increased levels of p53 target genes, e.g., PUMA and p21 (**Fig. 2I**). Diminished protein translation is a functional readout of nucleolar stress. The activity of mTOR signaling, which is important for protein synthesis, was inhibited as evidenced by decreased phosphorylation of mTOR^Ser2448^ and p70S6K^Thr389^ in MDA-MB-231 but not MDA-MB-453 SFCs (**Supplementary Fig. S6**).

### Dysregulated ribosome biogenesis is a characteristic feature of TNBC and can be used to predict patient prognosis

**Figure 3:**
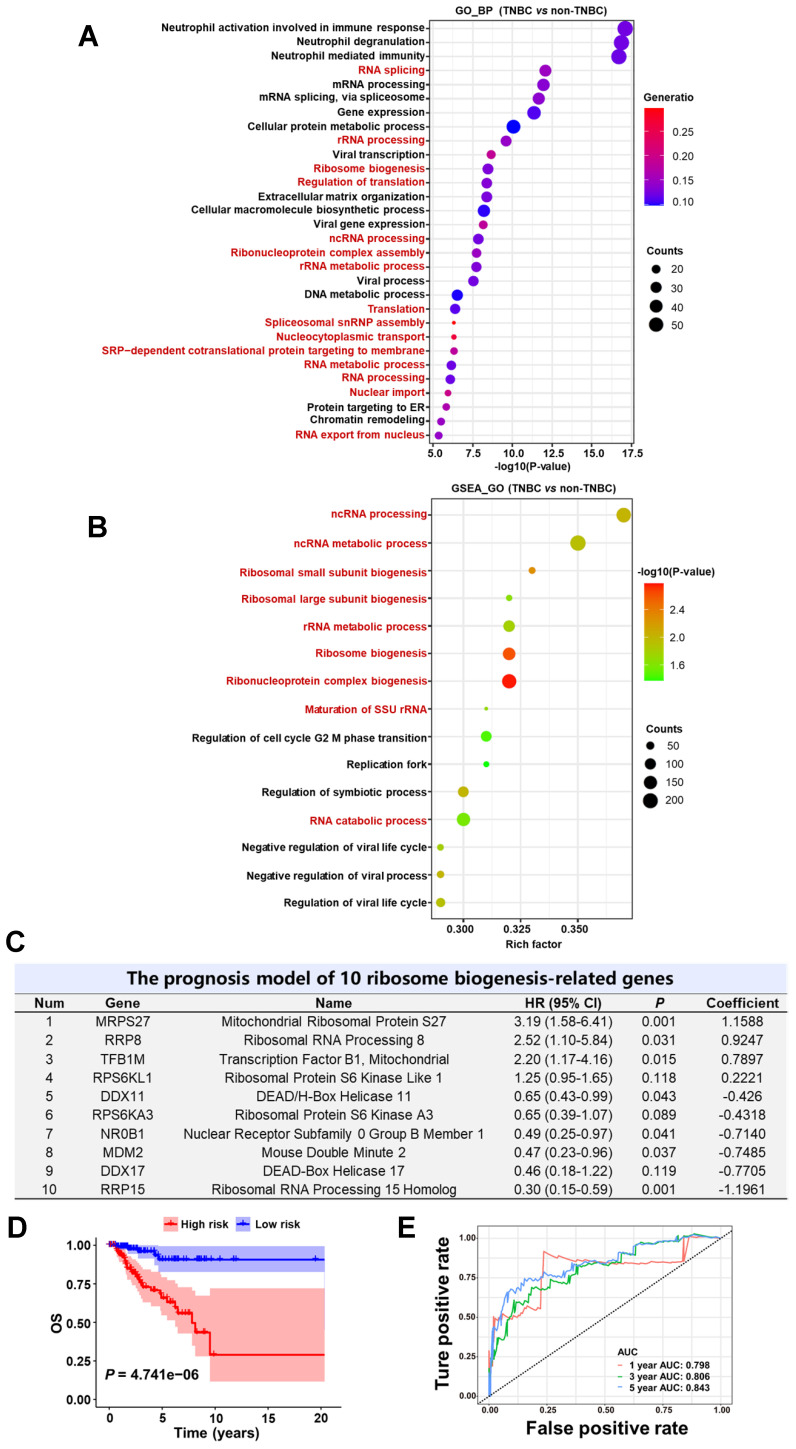
** Dysregulated ribosome biogenesis is a characteristic feature and can be used to predict prognosis in TNBC.** (**A and B**) Ribosome biogenesis as the major altered pathway (marked in red) in TNBC patient tissues revealed by GO analysis based on the iTRAQ proteomics data (**A**) and GSEA_GO analysis based on the TCGA-BRCA RNA-seq data (**B**). (**C**) The prognostic risk model comprising 10 ribosome biogenesis-related genes. (**D**) Kaplan-Meier survival analysis of TNBC patients in the high- and low-risk groups. (**E**) Time-dependent ROC curves of the model for 1-, 3-, and 5-year OS. BP, biological process; HR, hazard ratio; OS, overall survival; ROC, receiver-operating characteristic; AUC, area under the curve.

Given the above results demonstrating that lovastatin targets the ribosome biogenesis pathway in TNBC, we asked whether dysfunction of ribosome biogenesis is a characteristic feature in TNBC. In order to answer this question, we performed iTRAQ proteomics analysis on tissue samples of 40 breast cancer patients (10 TNBC *vs* 30 non-TNBC) from Hunan Cancer Hospital. GO biological process analysis revealed that among the top 30 enriched pathways, 15 were related to the ribosome biogenesis process, including RNA splicing, rRNA processing, ribosome biogenesis, regulation of translation, etc (**Fig. 3A**). GO cellular component and KEGG analyses confirmed the enrichment of ribosome biogenesis-related pathways in TNBC compared with non-TNBC tissues (data not shown).

We next analyzed the dataset obtained from TCGA-BRCA and performed Gene Set Enrichment Analysis (GSEA) to assess the enrichment of functional pathways based on the differentially expressed genes between TNBC and non-TNBC samples. In agreement with the results obtained from iTRAQ proteomics analysis of breast cancer tissues, GSEA_GO analysis of the TCGA-BRCA dataset revealed ribosome biogenesis-related pathways to be the major top altered categories in TNBC patient tissues (**Fig. 3B and Supplementary Fig. S7A**). GSEA_Reactome analysis also verified the enrichment of ribosome biogenesis-related pathways in TNBC patient tissues (**Supplementary Fig. S7B and C**). These results strongly suggest that dysregulation of ribosome biogenesis is a characteristic feature in TNBC compared with non-TNBC.

Given the above results that dysregulation of ribosome biogenesis is a characteristic feature in TNBC, we wondered whether a ribosome biogenesis-related gene signature with the prognostic value could be constructed to predict the outcomes of TNBC patients. Among the 548 ribosome biogenesis-related genes collected from MSigDB, more than 80% (451 genes) exhibited differential expression between TNBC and non-TNBC based on TCGA-BRCA dataset (**Supplementary Fig. S8A**). Through univariate Cox regression analysis, we identified 31 differentially expressed genes that contributed to the OS of TNBC patients (**Supplementary Fig. S8B**). Least absolute shrinkage and selection operator (LASSO) regression reduced the number of genes to 16 based on a minimum value of λ (**Supplementary Fig. S8C**). Through further multivariate Cox regression analysis, we identified a prognostic model based on 10 ribosome biogenesis-related genes (**Fig. 3C**). These genes included 4 risky genes (MRPS27, RRP8, TFB1M, RPS6KL1) and 6 protective genes (DDX11, RPS6KA3, NR0B1, MDM2, DDX17, RRP15). The subcellular localization and functions of these 10 genes are summarized in** Supplementary Table S6**. The role of MRPS27, the top-ranked risky gene, in mediating the effects of lovastatin in TNBC cells was further investigated (below).

We next evaluated the accuracy and reliability of the prognostic model in TNBC patients. Kaplan-Meier survival analysis revealed that patients of the high-risk group had significantly poorer OS compared with those of the low-risk group (*P* = 4.741e-06) (**Fig. 3D**). Consistently, the mortality of TNBC patients increased as the risk score increased (**Supplementary Fig. S8D**). Well separation of the expression levels of these 10 genes between the high- and low-risk TNBC patients was illustrated by the heatmap (**Supplementary Fig. S8E**). Time-dependent ROC curves revealed that the AUC values at 1-, 3-, and 5-year were 0.798, 0.806, and 0.843, respectively (**Fig. 3E**). These results indicate that the developed prognostic model has satisfactory predictive performance and exhibits potent power to predict survival of TNBC patients.

We then conducted validation using two external TNBC datasets (the GSE58812 cohort and the FUSCC cohort). Similar to the results obtained from the training set, the patients of the high-risk group had a shortened survival time compared with those of the low-risk group for the GSE58812 cohort (*P* = 0.049) (**Supplementary Fig. S9A**) and the FUSCC cohort (*P* = 0.003) (**Supplementary Fig. S9B**), respectively. Likewise, TNBC patients of the high-risk group were more likely to die compared with those in the low-risk group as demonstrated by the risk score curve and survival status scatter plots in the GSE58812 cohort (**Supplementary Fig. S9C**) and the FUSCC cohort (**Supplementary Fig. S9D**). Strong separation of the expression profiles of the 10 signature genes in patients of the high- and low-risk groups was demonstrated in the GSE58812 cohort (**Supplementary Fig. S9E**) and the FUSCC cohort (**Supplementary Fig. S9F**). Moreover, time-dependent ROC curves revealed that the AUC values at 1-, 3-, and 5-year were 0.889, 0.712, and 0.727 for the GSE58812 cohort (**Supplementary Fig. S9G**), respectively, and 0.718, 0.715, and 0.735 for the FUSCC cohort (**Supplementary Fig. S9H**), respectively. These results suggest that the prognostic model could robustly and accurately predict the prognosis of TNBC patients.

### Knockdown of MRPS27 induces nucleolar stress and inhibits translation efficiency of stemness-related pathways in TNBC

As shown above, mitochondrial ribosomal protein S27 (MRPS27) is the top-ranked risky gene in the prognostic signature which confers a 3.19-fold increase in hazard ratio in TNBC patients. We wondered whether MRPS27, a mitochondrially located protein, is also located outside the mitochondrion, exerting nucleolar-related functions. Interestingly, the HPA expression profile shows that in addition to the mitochondrial localization, MRPS27 is also located in the nucleolus (**Supplementary Fig. S10A**), providing a functional link of MRPS27 to the nucleolar-related functions inside the cell. In order to investigate the biological functions of MRPS27 in TNBC cells, we first performed GEPIA2 analysis to reveal the correlation between MRPS27 and nucleolar stress-related proteins. We found that MRPS27 was positively correlated with NPM and RPL3, the two nucleolar proteins targeted by lovastatin (**Supplementary Fig. S10B and C**).

**Figure 4:**
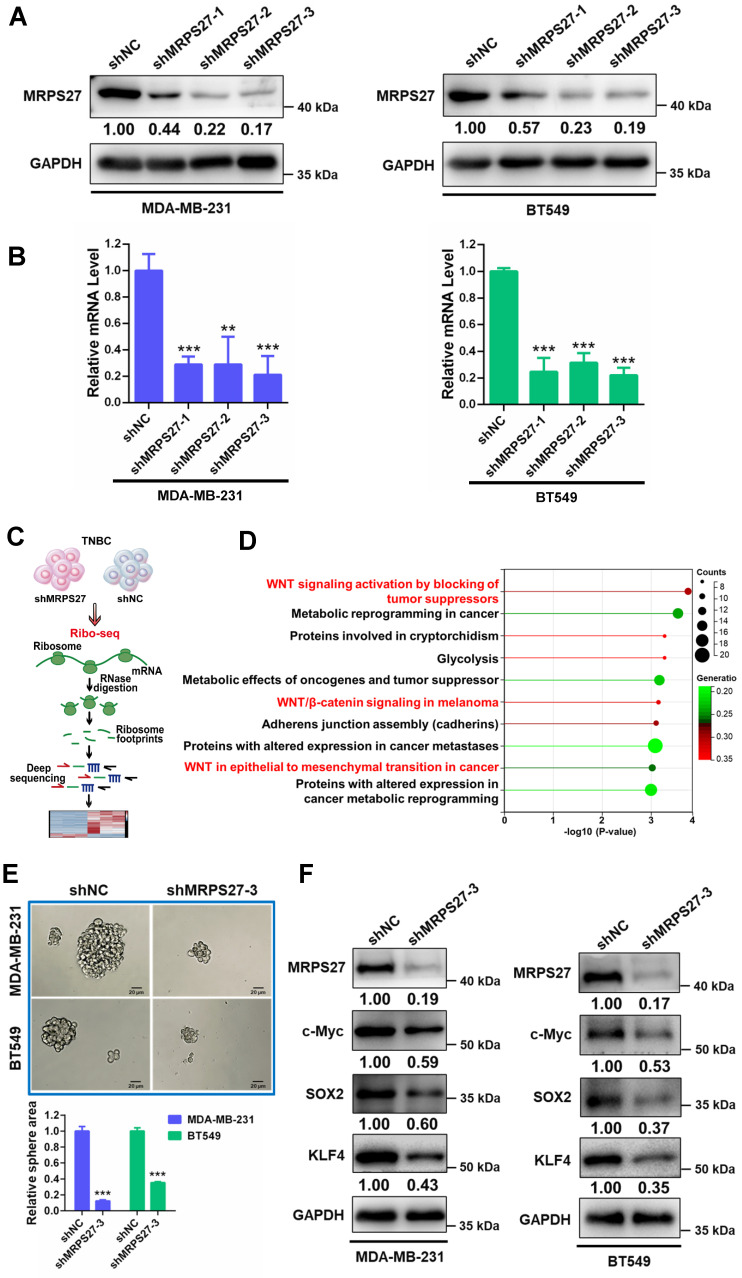
** Knockdown of MRPS27 inhibits the stemness properties of TNBC cells.** (**A and B**) Western blot (**A**) and qRT-PCR (**B**) analyses revealing the knockdown efficiency of MRPS27 in MDA-MB-231 and BT549 cells. (**C**) The flow chart for ribosome profiling in MRP27 knockdown TNBC cells and control cells. (**D**) Functional pathway analysis of differentially expressed genes identified from ribosome profiling in MRP27 knockdown MDA-MB-231 and BT549 cells compared with control cells. (**E**) Sphere formation of MDA-MB-231 and BT549 cells after knockdown of MRPS27 cultured in ultra-low attachment 6-well plates with serum-free stem cell medium. Scale bar = 20 μm. (**F**) Western blot analysis for stemness-related proteins after knockdown of MRPS27 in MDA-MB-231 and BT549 cells. **P < 0.01, ***P < 0.001.

We then asked whether MRPS27 is associated with the nucleolar stress response in TNBC cells. We generated MRPS27 stable knockdown TNBC cell lines (MDA-MB-231 and BT549) using three independent shRNAs. Knockdown of MRPS27 was confirmed using western blot analysis (**Fig. 4A**) and qRT-PCR (**Fig. 4B**). As anticipated, knockdown of MRPS27 decreased the transcript levels of the precursor 45S rRNA and the mature 28S, 5.8S, and 18S rRNAs in MDA-MB-231 and BT549 cells (**Supplementary Fig. S11**). Immunofluorescence-confocal microscopy demonstrated that MRPS27 knockdown also altered the protein level and the distribution of RPL3 in the cytoplasm and the nucleus of these cells (data not shown).

Next, we sought to determine the pathways regulated by MRPS27 in TNBC cells through ribosome profiling (Ribo-seq) (**Fig. 4C**). Interestingly, among the top 10 pathways regulated by MRPS27, 3 were related to Wnt/β-catenin signaling (**Fig. 4D**). The Wnt/β-catenin signaling pathway is known to be one of major pathways dysregulated in TNBC stemness 44. The correlation between MRPS27 and the stemness-related gene signature 45 was confirmed by GEPIA2 analysis in breast cancer patients (**Supplementary Fig. S12**). We further evaluated the effects of MRPS27 knockdown on stemness properties of TNBC cells and found that MRPS27 knockdown inhibited the sphere-forming activity (**Fig. 4E**) and decreased the levels of key stemness-related proteins including c-Myc, SOX2, and KLF4 (**Fig. 4F**) in both MDA-MB-231 and BT549 cells.

**Figure 5:**
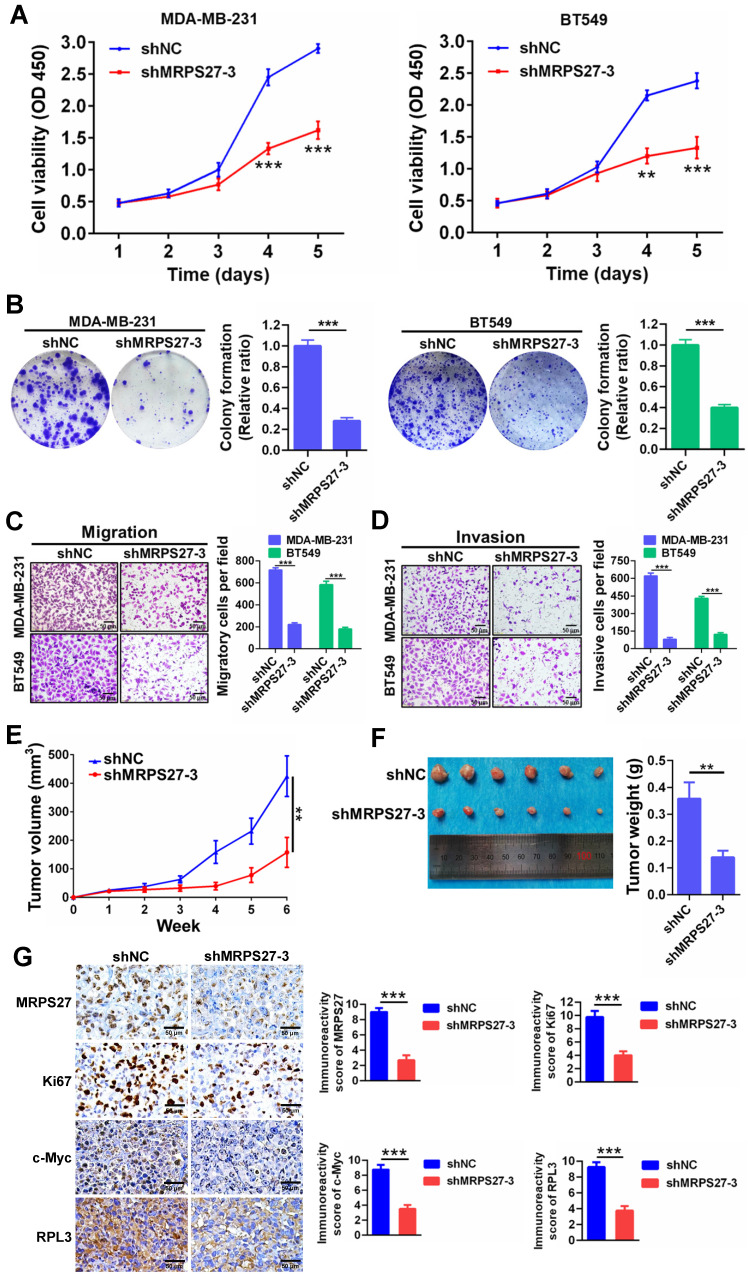
** Knockdown of MRPS27 inhibits the malignant phenotypes of TNBC cells *in vitro* and *in vivo*.** (**A**) CCK8 assay for cell viability after knockdown of MRPS27 in MDA-MB-231 and BT549 cells. (**B**) Colony formation assay for the number of colonies after knockdown of MRPS27 in MDA-MB-231 and BT549 cells. (**C and D**) Transwell migration (**C**) and invasion (**D**) assays for migratory and invasive abilities after knockdown of MRPS27 in MDA-MB-231 and BT549 cells. Scale bar = 50 μm. (**E**) Growth curves of TNBC orthotopic tumors derived from MRPS27 knockdown MDA-MB-231 cells compared with the control cells. (**F**) Weights of TNBC orthotopic tumors derived from MRPS27 knockdown MDA-MB-231 cells compared with the control cells. (**G**) Representative immunohistochemical staining images for MRPS27, Ki67, c-Myc, and RPL3 in MRPS27 knockdown MDA-MB-231 cell- *vs* control cell-derived orthotopic tumors. Scale bar = 50 μm. **P < 0.01, ***P < 0.001.

Additionally, knockdown of MRPS27 resulted in inhibition of cell proliferation as revealed by decreases in cell viability (**Fig. 5A**) and colony formation (**Fig. 5B**). Knockdown of MRPS27 also inhibited migratory and invasive abilities of MDA-MB-231 and BT549 cells in transwell migration (**Fig. 5C**) and invasion (**Fig. 5D**) assays. We then used a mouse model of orthotopic tumor growth to evaluate the effect of MRPS27 knockdown *in vivo*. Compared with those injected with shNC cells, the mice receiving MRPS27 knockdown cells had decreased tumor growth over a period of 6 weeks post injection (**Fig. 5E**) and decreased tumor weight (**Fig. 5F**). Consistently, the protein levels of Ki67 (a proliferation marker), c-Myc (a stemness marker), and RPL3 (a ribosomal protein) were decreased in the tumor of mice injected with shMRPS27 knockdown MDA-MB-231 cells compared with the control cells (**Fig. 5G**). Collectively, these results suggest that MRPS27 predominantly regulates translation efficiency of stemness-related pathways in TNBC.

### Overexpression of MRPS27 attenuates the stemness-inhibitory effect of lovastatin in TNBC cells

**Figure 6:**
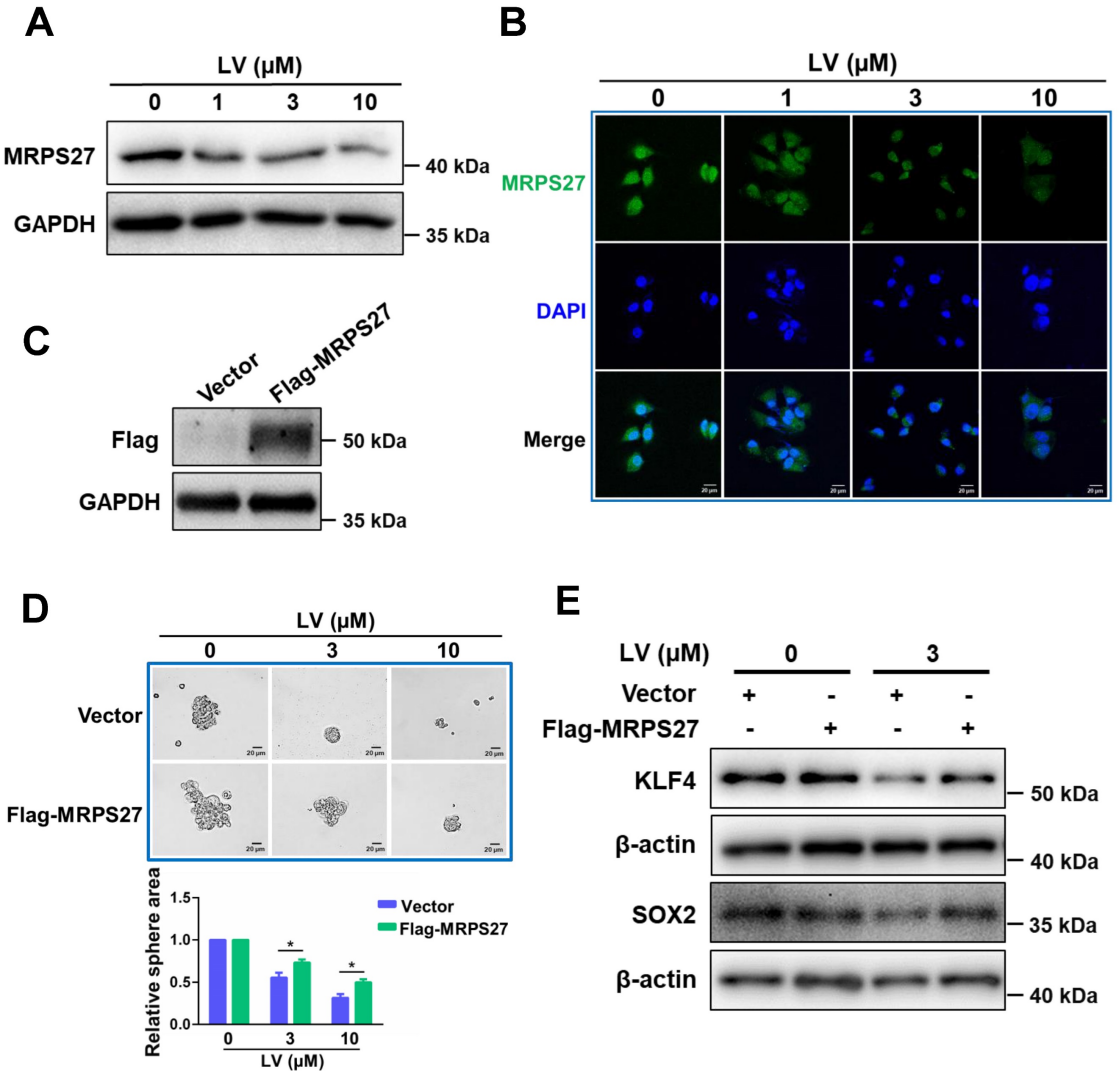
** Overexpression of MRPS27 attenuates the stemness inhibitory effect of lovastatin in TNBC cells.** (**A and B**) Western blot (**A**) and immunofluorescence-confocal microscopy (**B**) analyses revealing the protein level of MRPS27 in MDA-MB-231 cells after treatment with different concentrations of lovastatin. (**C**) Western blot analysis showing the overexpression efficiency of MRPS27 in HCC1937 cells. (**D**) Sphere formation in MRPS27-overexpressing or vector control HCC1937 cells after treatment with different concentrations of lovastatin. Scale bar = 20 μm. Bottom, quantifications of the sphere area. (**E**) Western blot analysis of stemness-related proteins in HCC1937 cells overexpressing MRPS27 or control cells treated with or without lovastatin. *P < 0.05; LV, lovastatin.

In order to investigate whether the stemness-inhibitory effect of lovastatin in TNBC cells was mediated by MRPS27, we first performed western blot analysis to determine the protein level of MRPS27 in TNBC cells treated with various concentrations of lovastatin. We found that lovastatin downregulated the protein level of MRPS27 in MDA-MB-231 cells in a dose-dependent manner (**Fig. 6A**). We further validated MRPS27 downregulation by lovastatin using immunofluorescence-confocal microscopy (**Fig. 6B**). Then, we generated an MRPS27-overexpressing TNBC cell line (HCC1937) by transient transfection to investigate whether overexpression of MRPS27 could reverse the stemness-inhibitory effect of lovastatin. Overexpression of MRPS27 was confirmed using western blot analysis (**Fig. 6C**). Indeed, overexpression of MRPS27 attenuated the inhibitory effect of lovastatin on stemness properties in TNBC cells as evidenced by reversal of the inhibition of sphere formation (**Fig. 6D**) and stemness-related markers (**Fig. 6E**).

### MRPS27 is highly expressed and correlated with the clinicopathologic features in TNBC patients

**Figure 7:**
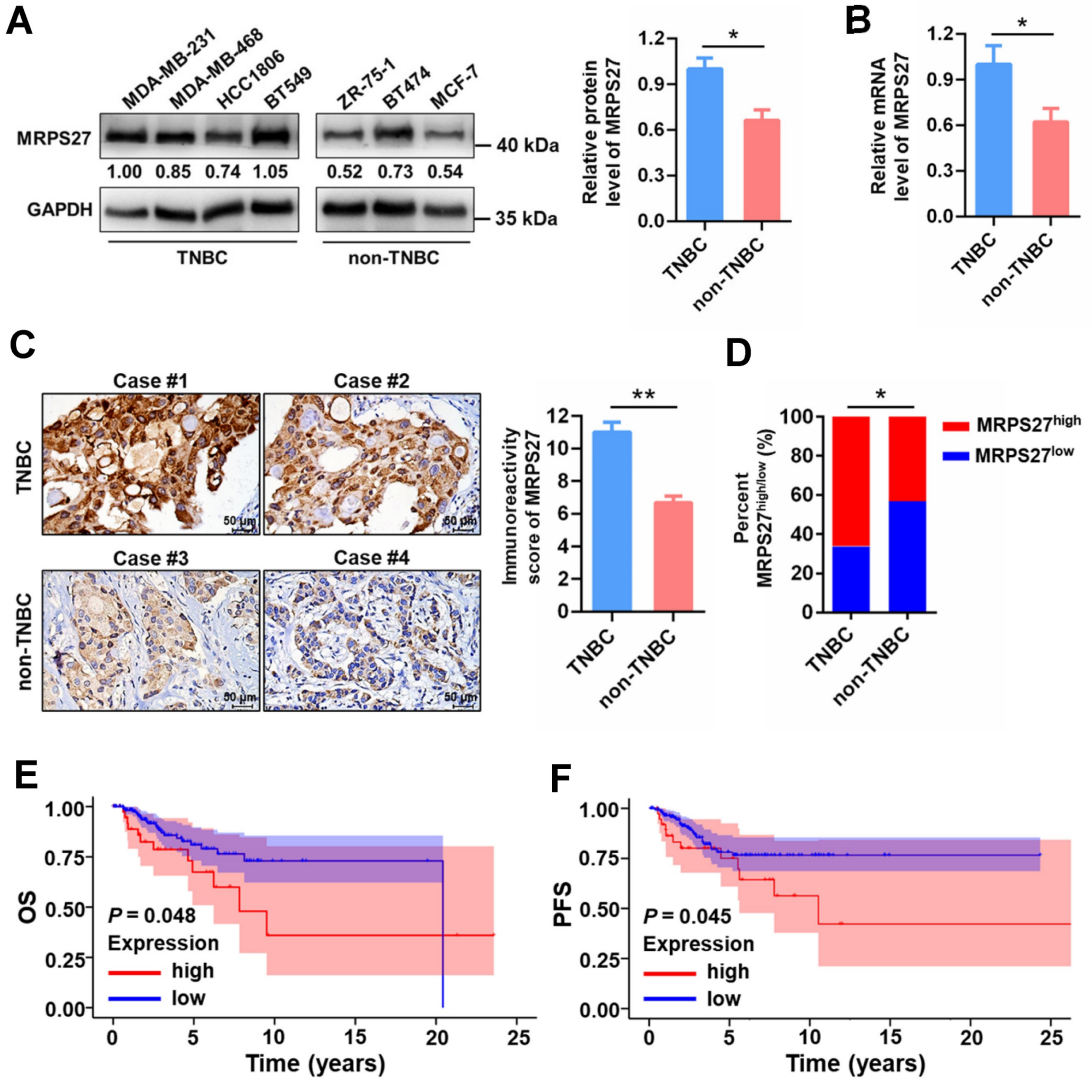
** MRPS27 is expressed at a higher level and correlated with poor prognosis in TNBC patients.** (**A**) The protein levels of MRPS27 in TNBC and non-TNBC cell lines. Right, quantifications of the protein level of MRPS27. (**B**) The mRNA levels of MRPS27 in TNBC and non-TNBC cell lines. (**C**) Representative immunohistochemical staining images for MRPS27 in TNBC and non-TNBC patient tissues. (**D**) Chi-square analysis of the distribution of MRPS27^high^ *vs* MRPS27^low^ cases between TNBC and non-TNBC patient tissues. (**E and F**) Kaplan-Meier survival analysis revealing the relationship between the expression of MRPS27 and OS (**E**) or PFS (**F**) in TNBC patients based on the TCGA-BRCA dataset. *P < 0.05, **P < 0.01; OS, overall survival; PFS, progression-free survival.

To reveal the pathophysiological relevance of MRPS27 in TNBC, we performed bioinformatics and experimental analyses on the expression profile of MRPS27 in cancer tissues and cell lines. Pan-cancer analysis of the Human Protein Atlas (HPA) database revealed that among the various cancer types expressing MRPS27, breast cancer was the third-ranked cancer type with the majority (>90%) of patients appearing positive for MRPS27 (**Supplementary Fig. S13**). In addition, the protein level of MRPS27 was higher in breast cancer compared with normal tissues (data not shown). We further found that the expression of MRPS27 was higher in TNBC compared with non-TNBC cell lines at the protein level (*P* < 0.05) (**Fig. 7A**) and the mRNA level (*P* < 0.05) (**Fig. 7B**). Immunohistochemistry revealed that MRPS27 was expressed at a higher level in TNBC compared with non-TNBC patient tissues (**Fig. 7C**). Chi-square analysis of the MRPS27 expression level confirmed the presence of a significantly higher proportion of MRPS27^high^ cases in the TNBC group compared with the non-TNBC group, which contained a higher proportion of MRPS27^low^ cases (**Fig. 7D**).

In addition, MRPS27 expression was positively correlated with stage (*P* = 0.039) and lymph node involvement (*P* = 0.045) in TNBC patients (**Supplementary Table S7**). Kaplan-Meier plotter analysis revealed that higher expression of MRPS27 was associated with poorer OS (**Fig. 7E**) and progression-free survival (PFS) (**Fig. 7F**) in TNBC patients. These results suggest that MRPS27 is expressed at higher level in TNBC patient tissues and that higher expression of MRPS27 is correlated with unfavorable outcomes of TNBC patients.

## Discussion

In this study, we present strong evidence to demonstrate that dysregulated ribosome biogenesis is a characteristic feature and is a targetable vulnerability in TNBC. Previously, Belin *et al*. revealed that dysregulation of ribosome biogenesis is observed in a variety of cancer types including breast cancer 46. To support the pivotal role of ribosome biogenesis dysregulation in TNBC, the genes encoding the RNA polymerase I subunits, which are responsible for the transcription of rRNAs in the nucleolus, are found to be highly expressed in TNBC compared to non-TNBC tissue samples. Furthermore, higher expression levels of these genes are associated with poorer prognosis in breast cancer patients (**Supplementary Fig. S14**). Our observations are in agreement with a previous report that a higher nucleolar score predicts poorer breast cancer-specific survival in a large cohort of breast cancer patients 47. Moreover, it has been demonstrated that induction of ribosome biogenesis is a general feature of the epithelial-to-mesenchymal transition (EMT) program and ribosome biogenesis during cell cycle fuels EMT in breast cancer cells 48, further justifying targeting the ribosome biogenesis pathway as an innovative therapeutic strategy for TNBC.

A plethora of studies have demonstrated that inducing nucleolar stress through targeting the ribosome biogenesis pathway can be used as an innovative strategy for cancer therapy. Oxaliplatin, one of the platinum compounds that is used as a first-line treatment for several types of cancer including breast cancer, has been shown to eradicate cancer cells by inducing cytotoxicity through targeting the ribosomal protein RPL11 49. CX-5461, a potent selective and orally available inhibitor of RNA polymerase I, exhibits anti-cancer activity in preclinical models, particularly in Myc-driven malignancies 50. A multicenter phase I trial of CX-5461 was recently completed (clinical trial ID: NCT02719977) in solid tumors including advanced breast cancer with well-tolerated adverse events 51. Several other compounds that inhibit cancer cells by inducing nucleolar stress have entered clinical trials and some of them have been approved for clinical use 52. One major advantage of the agents that selectively target the nucleolus without causing DNA damage could be that they are less toxic to normal cells which require less ribosome biogenesis compared to cancer cells.

As a widely prescribed lipid-lowering drug, the anti-cancer activity of lovastatin has attracted intensive research interest 29. Compared with the traditional way of developing novel therapeutics, drug repurposing is a safe, cost-effective, and time-saving approach to more efficient cancer therapy 53. However, none of the approved drugs with acceptable toxicity profile and CSC-targeting activity have been thoroughly evaluated in TNBC. Here, we present data to demonstrate that lovastatin has the potential of targeting the ribosome biogenesis pathway for innovative TNBC therapy. Induction of nucleolar stress by lovastatin was demonstrated by its ability to induce translocation of nucleolar proteins, such as NPM and NOLC1, and the ribosomal proteins, such as RPL3 and RPS10, inhibition of rRNA levels, and increase in p53 transcriptional activity.

NPM and NOLC1 belong to the family of intrinsically disordered proteins (IDPs) that play an important role in the formation of membraneless organelles such as the nucleolus 12, 54. Recently, targeting the IDP family proteins has been explored as a novel strategy for breast cancer 55. NONO/p54nrb is an IDP located in one of the nuclear compartments known as the paraspeckle, which is involved in a wide range of transcriptomic regulatory activities 56, 57. We recently explored the therapeutic value of NONO in TNBC and found that while higher expression level of NONO was associated with poorer OS in breast cancer patients, siRNA-mediated silencing of NONO significantly inhibited TNBC tumor growth *in vivo* 58. Hopefully, inducing nucleolar stress in TNBC *via* targeting the lysine-rich IDPs will shed light on the therapy of this type of hard-to-treat malignancy.

Interestingly, two of the four risky genes in the ribosome biogenesis-related gene signature, namely, MRPS27 and mitochondrial transcription factor B1 (TFB1M) are associated with mitochondrial functions. On the one hand, as a component of the small subunit of the mitochondrial ribosome, MRPS27 plays a fundamental role in the formation of physical bridges with the large subunit 59. On the other hand, as a member of the pentatricopeptide repeat (PPR) domain family proteins, MRPS27 serves as an RNA-binding protein that associates the 12S mitochondrial rRNA and is involved in the translation of mRNAs important for mitochondrial functions 60. Therefore, knockdown of MRPS27 is expected to have significant impact on structural and functional aspects of the mitochondrion, which is beyond the scope of the current study. Nevertheless, we show that MRPS27 is positively correlated with the stemness-related gene signature in breast cancer (**Supplementary Fig. S12**) and MRPS27 knockdown induces nucleolar stress in TNBC (**Supplementary Fig. S11**). Considering the important roles played by MRPS27 in mitochondrial structure and function, it is highly believed that MRPS27 knockdown-induced mitochondrial dysfunction might be involved in nucleolar stress in TNBC cells.

In contrast, TFB1M (also called mitochondrial 12S rRNA dimethylase 1) is a methyltransferase that specifically methylates two consecutive adenine residues within the stem-loop of the 12S mitochondrial rRNA 61. Both of these two molecules play an important role in the assembly of the complete mitochondrial ribosome followed by the synthesis of mitochondrial proteins 62, and therefore, their dysfunction may cause undesirable cellular consequences. Besides the known function in the synthesis of the enzymes required for proper mitochondrial function, other functions of the ribosomal proteins are poorly characterized. Here, we present data to show that MRPS27 plays a crucial role in maintaining the stemness properties and underlies the CSC-inhibitory effect of lovastatin in TNBC. This broadens our prospect of the functions of the mitochondrial ribosomal proteins besides their canonical mitochondrial functions. Whether MRPS27 can be exploited as a *bona fide* therapeutic target for TNBC needs to be further explored.

In summary, our results demonstrate that dysregulation of ribosome biogenesis is a characteristic feature in TNBC, which underlies the sensitivity of TNBC CSCs to lovastatin. Identification of dysregulated ribosome biogenesis as a therapeutic vulnerability in TNBC opens new areas to explore for innovative therapeutic strategies for TNBC.

## Supplementary Material

Supplementary figures and tables.
