# Supplementary material for: Dysregulated Ribosome Biogenesis Is a Targetable Vulnerability in Triple-Negative Breast Cancer: MRPS27 as a Key Mediator of the Stemness-inhibitory Effect of Lovastatin
Source: Int J Biol Sci. 2024 Mar 18;20(6):2130–48. doi: 10.7150/ijbs.94058 (PMC11008279; doi:10.7150/ijbs.94058)
Supplement: Supplementary file 1 — Supplementary figures and tables. [file ijbsv20p2130s1.pdf]

Dysregulated Ribosome Biogenesis Is a Targetable Vulnerability in Triple-Negative Breast Cancer: MRPS27 as a Key Mediator of the Stemness-inhibitory Effect of Lovastatin

Running title: Ribosome Biogenesis as a Targetable Vulnerability in TNBC

Chanjuan Zheng,[1,2] Hui Yao,[1,2] Lu Lu,[1,2] Hongqi Li,[3] Lei Zhou,[3] Xueyan He,[3] Xi Xu,[1,2] Hongzhuo Xia,[1,2] Siyu Ding,[1,2] Yiyuan Yang,[1,2] Xinyu Wang,[1,2] Muyao Wu,[1,2] Lian Xue,[1,2] Sisi Chen,[1,2] Xiaojun Peng,[4] Zhongyi Cheng,[4] Yian Wang,[1,2] Guangchun He,[1,2] Shujun Fu,[1,2] Evan T. Keller,[5] Suling Liu,[3]* Yi-zhou Jiang, [6,7]* and Xiyun Deng[1,2]*

SUPPLEMENTARY FIGURES 1-14 and TABLES 1-7

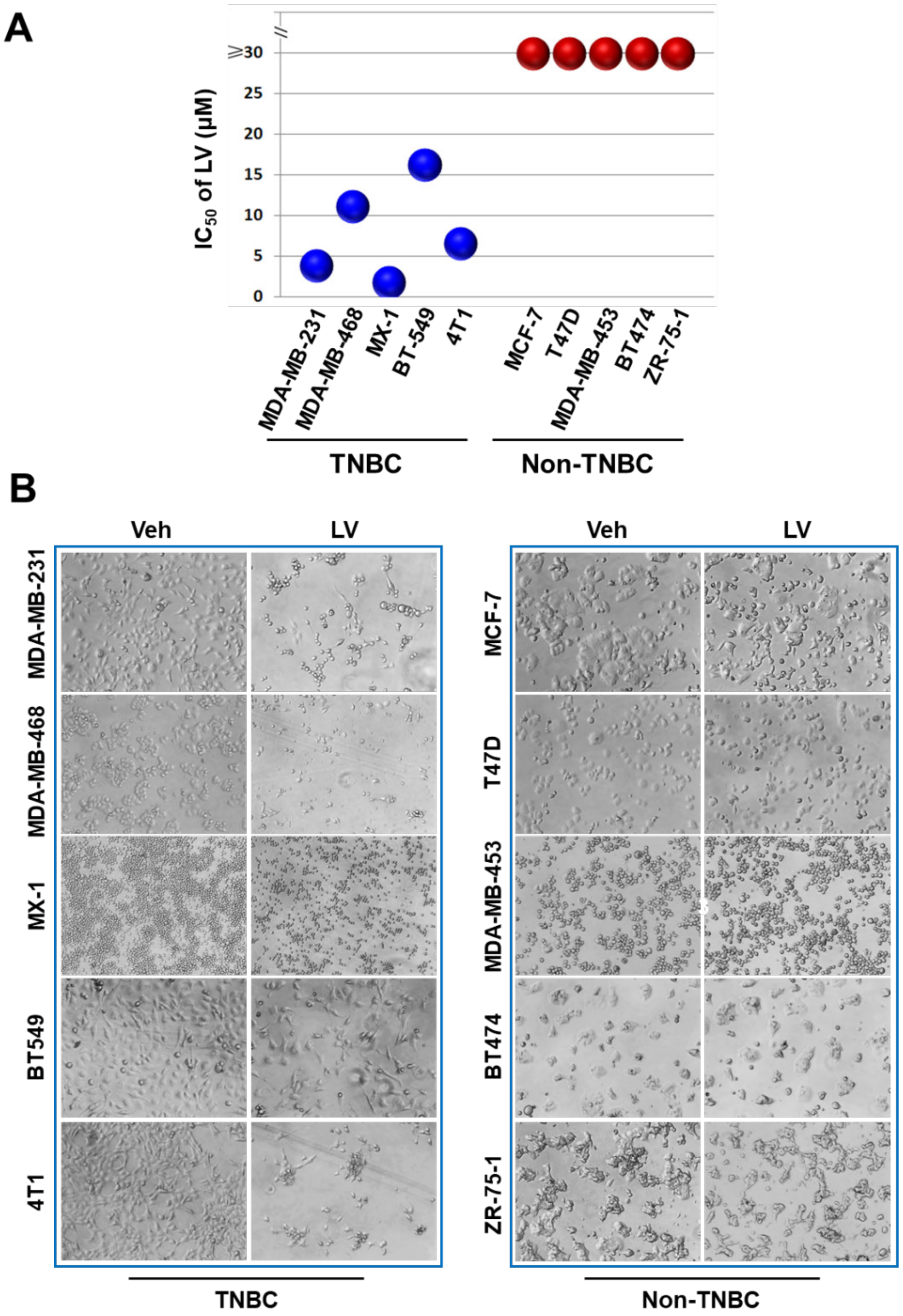

Supplementary Figure S1. Preferential inhibition of TNBC compared with non-TNBC cell lines by lovastatin.

(**A**) IC_{50} values of lovastatin calculated from cell viability assay on TNBC and non-TNBC cell lines treated with different concentrations of lovastatin. For simplicity, a maximal IC_{50} of 30 μM was plotted for those with an $IC_{50} \geq 30$ μM. (**B**) Representative photomicrographic images showing the effect of lovastatin (48 h, 10 μM) on cell morphology of TNBC *vs* non-TNBC cells.

Veh, vehicle; LV, lovastatin.

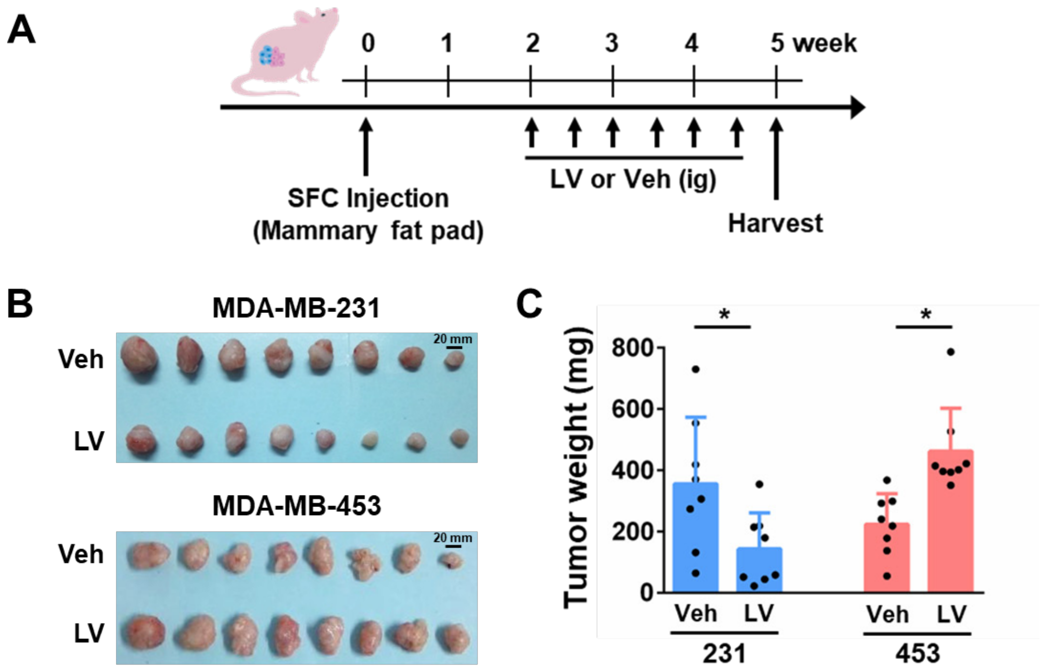

Supplementary Figure S2. Lovastatin inhibits TNBC cancer stem cells *in vivo*.

(**A**) Schematic of the mouse model of orthotopic tumor growth. (**B**) Images of the orthotopic tumors resected from MDA-MB-231 and MDA-MB-453 SFCs in nude mice after lovastatin or vehicle treatment. n = 8 mice per group. (**C**) Average tumor weights at the end of treatments. Data are shown as mean ± SEM.

*$P < 0.05$, Veh, vehicle; LV, lovastatin; SFCs, sphere-forming cells; ig: intragastric administration.

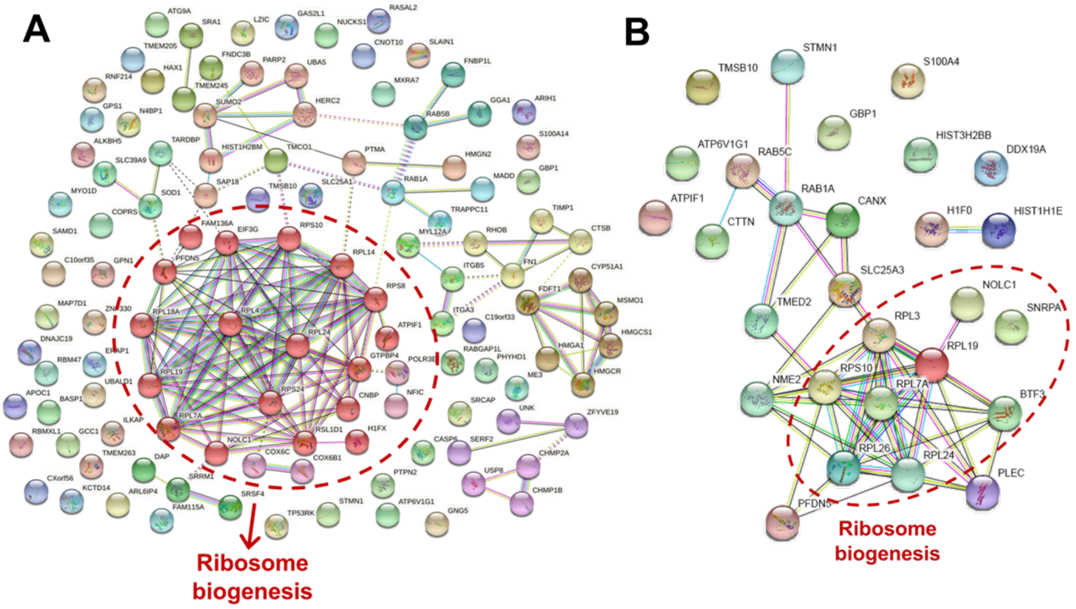

Supplementary Figure S3. PPI network for differentially regulated proteins in lovastatin-treated MDA-MB-231 cancer stem cells.

(**A**) PPI network generated from the proteomics profile showing the enrichment of the ribosome biogenesis pathway in lovastatin-treated MDA-MB-231 SFCs. (**B**) PPI network generated from the lysine crotonylation profile showing the enrichment of the ribosome biogenesis pathway in lovastatin-treated MDA-MB-231 SFCs.

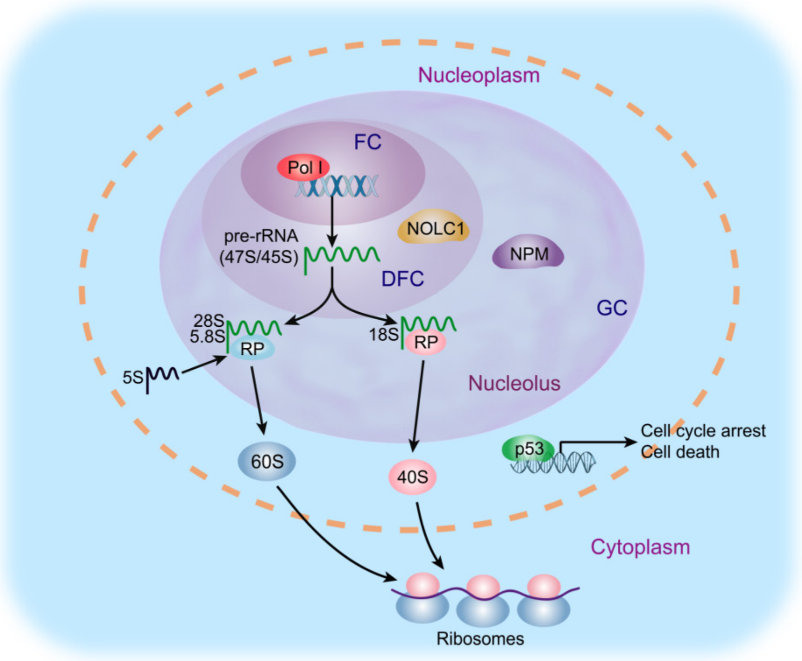

Supplementary Figure S4. The ribosome biogenesis pathway targeted by lovastatin.

The actions of lovastatin exerted on the ribosome biogenesis pathway include: 1) translocation of nucleolar proteins (NPM and NOLC1), 2) inhibition of the precursor 47S/45S rRNAs and the mature rRNAs (18S, 28S, 5.8S), and 3) increased intracellular level of p53 followed by increased transcription of its target genes.

FC, fibrillar center; DFC, dense fibrillar component; GC, granular component; Pol I, polymerase I; NOLC1, nucleolar and coiled-body phosphoprotein 1; NPM, nucleophosmin; RP, ribosomal protein.

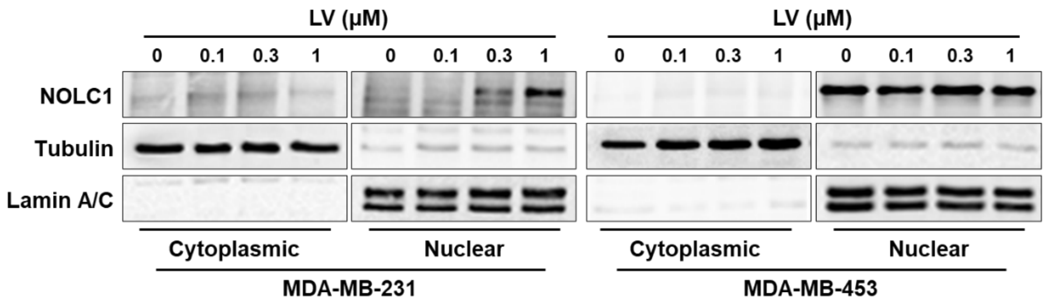

Supplementary Figure S5. Lovastatin increases the protein level of NOLC1 in the nucleus of TNBC cancer stem cells.

Western blot analysis of NOLC1 in the cytoplasmic and nuclear fractions of MDA-MB-231 and MDA-MB-453 SFCs. Tubulin and lamin A/C were used as loading controls for the cytoplasmic and nuclear lysates, respectively.

LV, lovastatin.

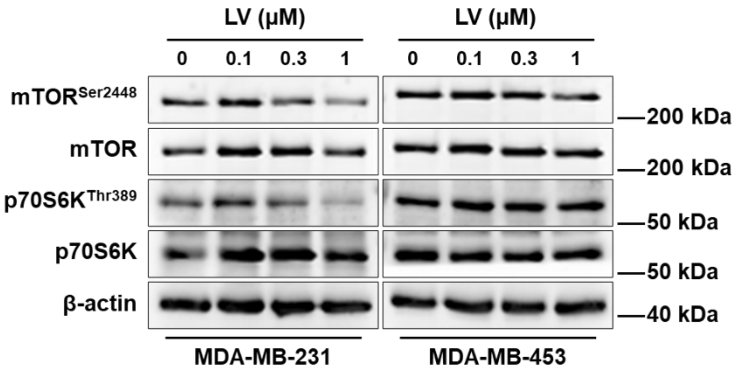

Supplementary Figure S6. Lovastatin inhibits the protein translation pathway in TNBC cancer stem cells.

Western blot analysis for phosphorylated and total mTOR and p70S6K in MDA-MB-231 and MDA-MB-453 SFCs after treatment with different concentrations of lovastatin.

LV, lovastatin.

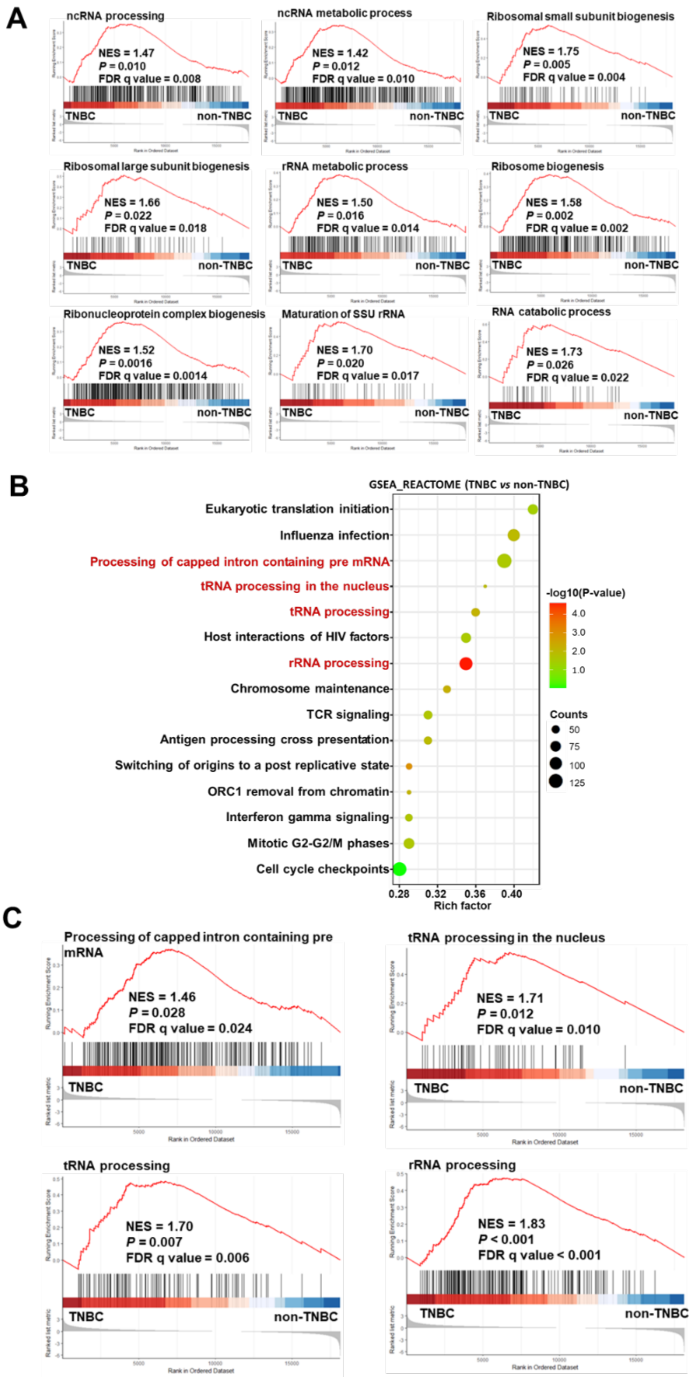

Supplementary Figure S7. Enrichment of ribosome biogenesis-related pathways as a characteristic feature in TNBC patient tissues.

(**A**) GSEA significant enrichment plots of ribosome biogenesis-related pathways identified from TCGA-BRCA database in TNBC patients. (**B**) Enrichment of ribosome biogenesis-related pathways (marked in red) in TNBC patients revealed by GSEA_Reactome analysis based on TCGA-BRCA RNA-seq data. (**C**) GSEA_Reactome significant enrichment plots of ribosome biogenesis-related pathways in TNBC patients.

NES, normalized enrichment score; FDR, false discovery rate.

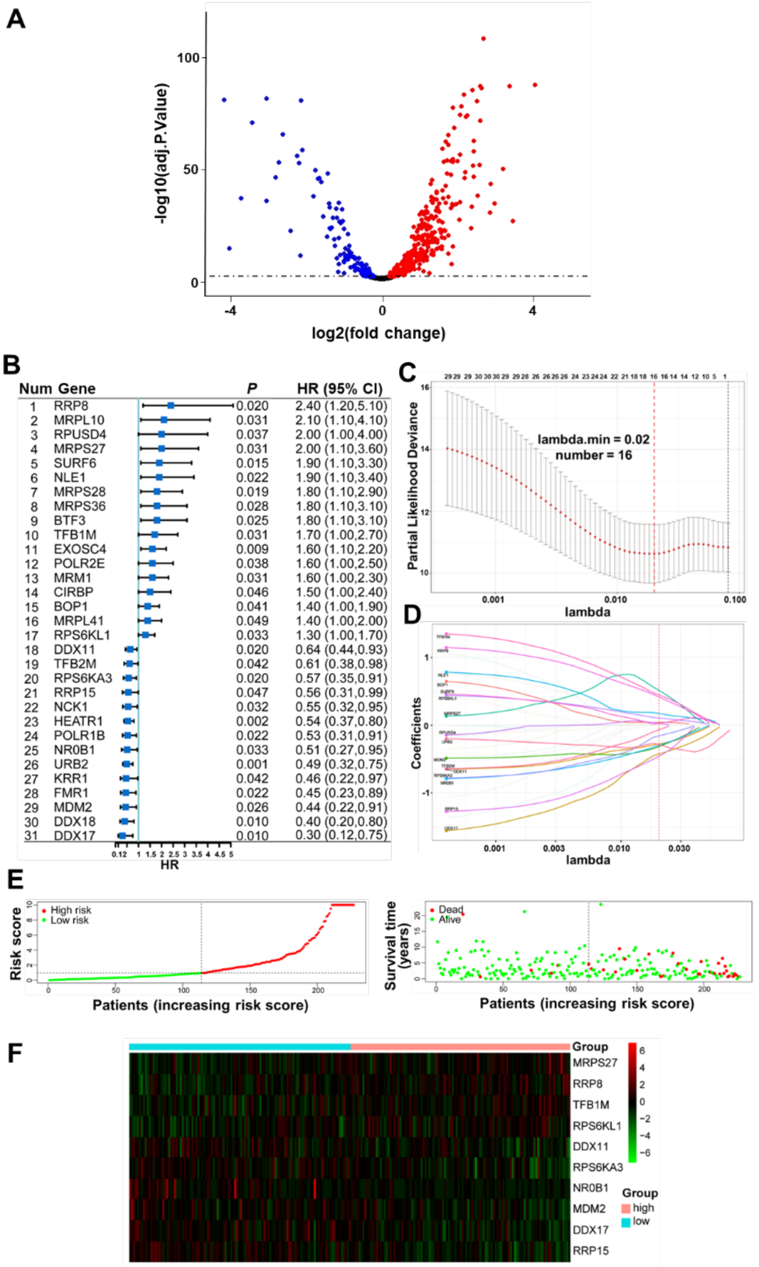

Supplementary Figure S8. Construction and evaluation of a prognostic model of TNBC patients based on 10 ribosome biogenesis-related genes in the training set.

(**A**) Volcano plot of differentially expressed ribosome biogenesis-related genes in TNBC compared with non-TNBC patient tissues in the TCGA-BRCA dataset. (**B**) Univariate Cox regression analysis of differentially expressed ribosome biogenesis-related genes associated with OS in TNBC patients. (**C**) Penalty parameter λ selection in the LASSO model using 10-fold cross-validation *via* minimum criteria. (**D**) LASSO coefficient spectrum of ribosome biogenesis-related genes enrolled in the model. (**E and F**) Distribution of the risk score, survival status (**E**), and heatmap of the expression profiles (**F**) of the 10 ribosome biogenesis-related genes between the high- and low-risk TNBC patients.

LASSO, least absolute shrinkage and selection operator.

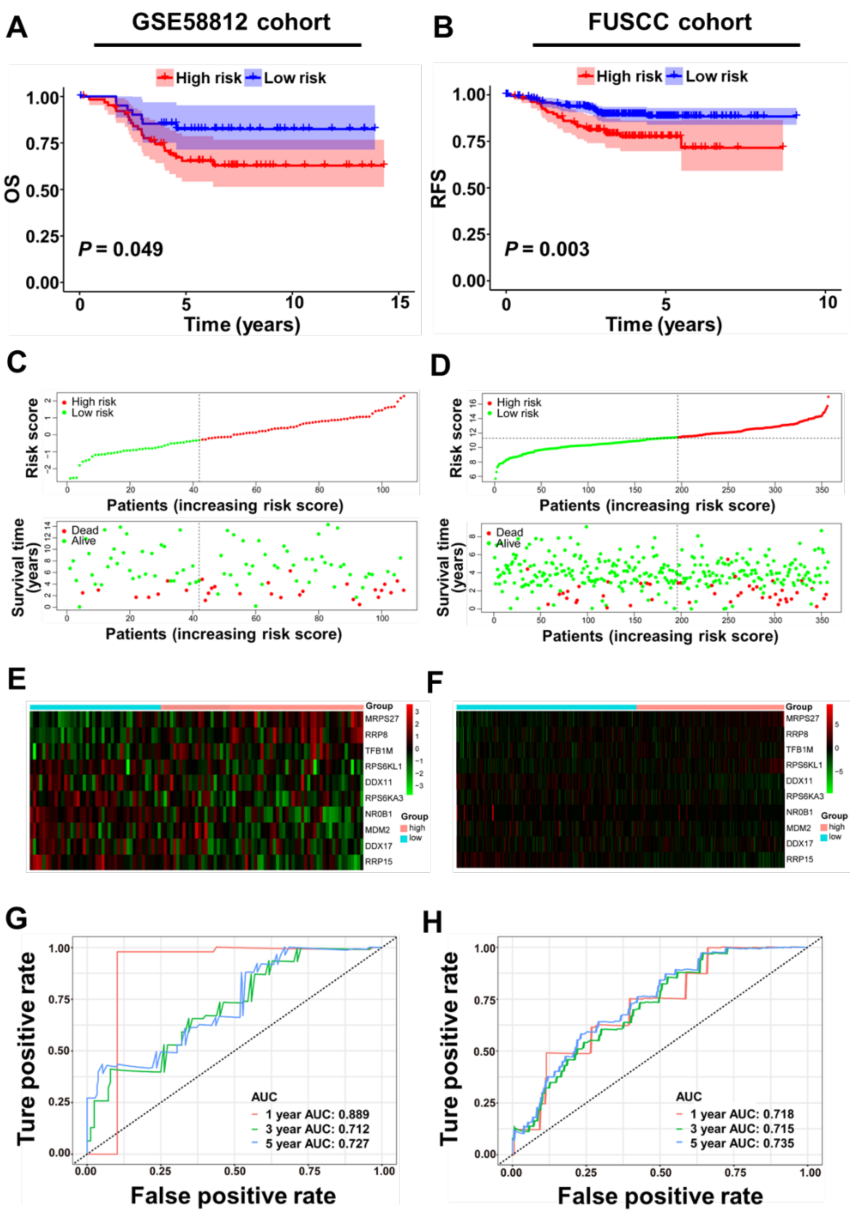

Supplementary Figure S9. Validation of the prognostic model of TNBC patients in external datasets (GSE58812, FUSCC).

(**A** and **B**) Kaplan-Meier survival analysis of TNBC patients in the high- and low-risk groups based on the prognostic model in the GSE58812 (**A**) and the FUSCC (**B**) cohorts, respectively. (**C** and **D**) Distribution of risk score and survival status between the high- and low-risk TNBC patients for GSE58812 (**C**) and FUSCC (**D**). (**E** and **F**) Heatmap of the expression of the 10 ribosome biogenesis-related genes between the high- and low-risk TNBC patients for GSE58812 (**E**) and FUSCC (**F**). (**G** and **H**) Time-dependent ROC curves of the model for 1-, 3-, and 5-year OS for GSE58812 (**G**) and FUSCC (**H**).

OS, overall survival; RFS, relapse-free survival; ROC, receiver-operating characteristic; AUC, area under the curve.

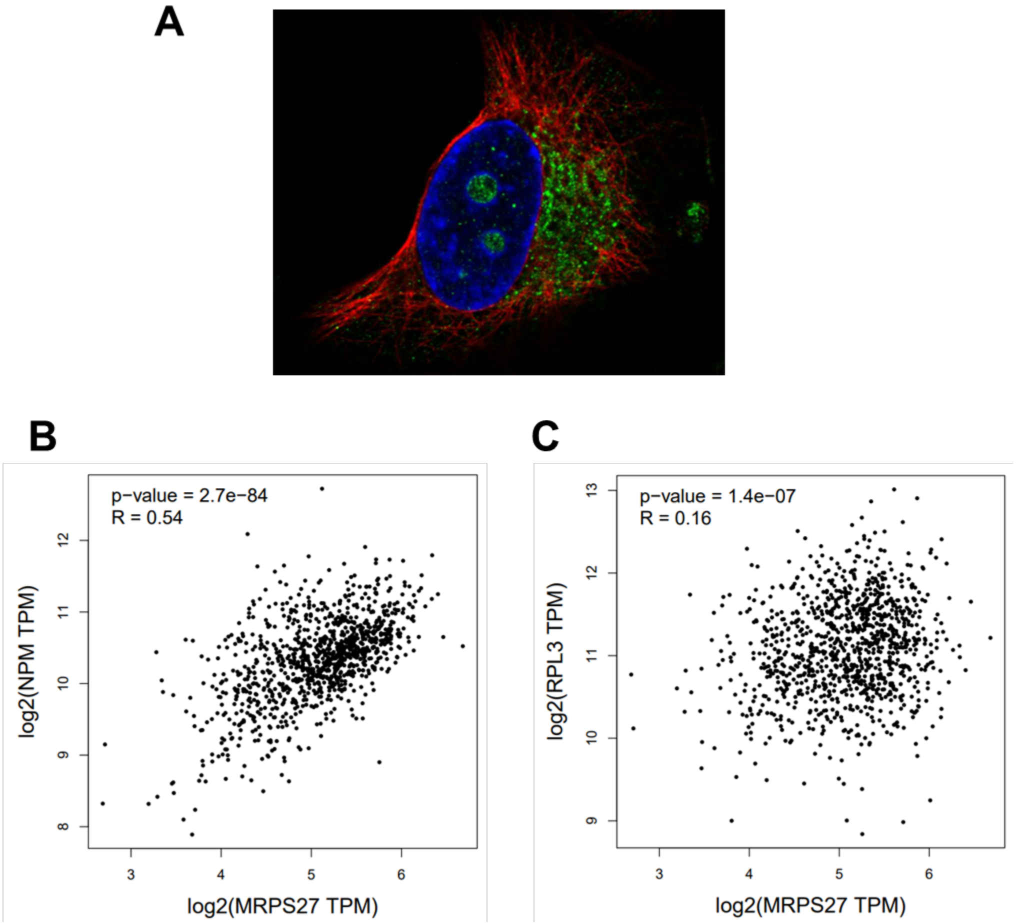

Supplementary Figure S10. The mitochondrial ribosomal protein MRPS27 is also located in the nucleolus and correlated with nucleolar stress-related proteins.

(**A**) HPA expression profile shows that MPRS27 is located in the mitochondrion and the nucleolus. (**B** and **C**) Correlation between MRPS27 and nucleolar stress-related proteins NPM (**B**) and RPL3 (**C**) in breast cancer analyzed by GEPIA2 database.

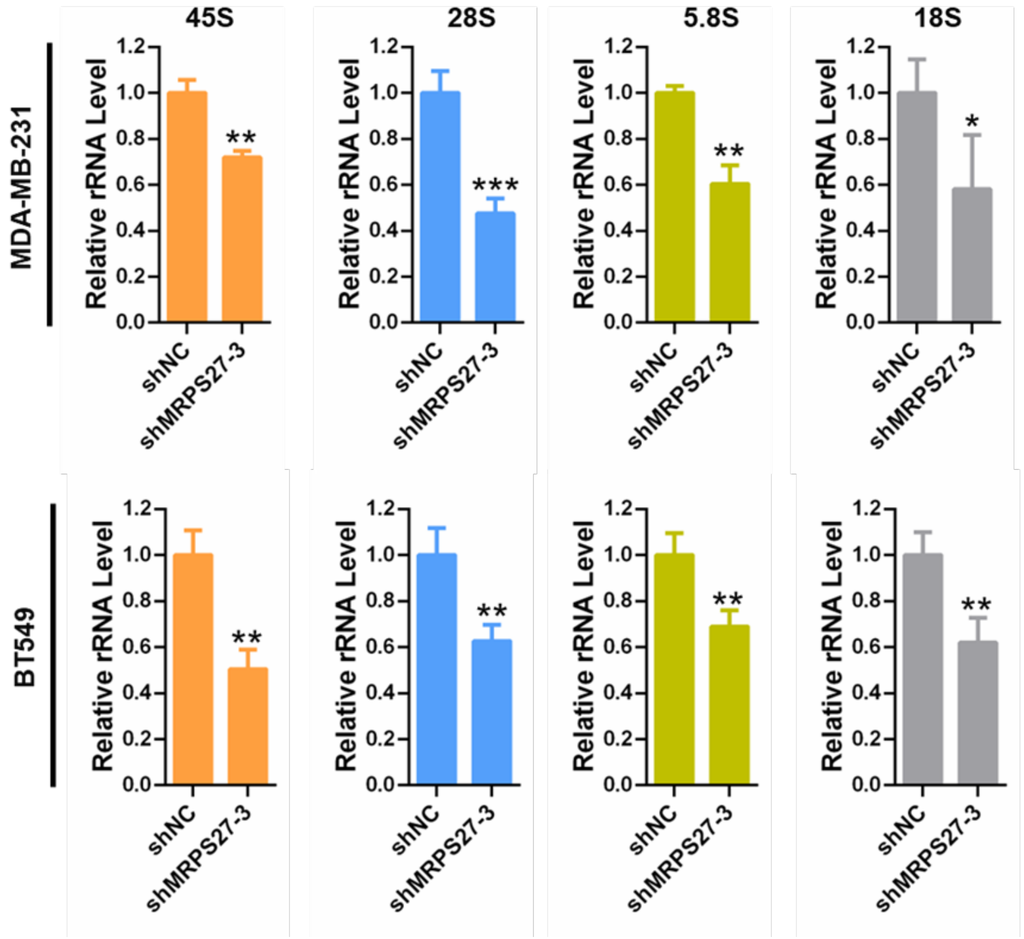

Supplementary Figure S11. Knockdown of MRPS27 induces nucleolar stress in TNBC cells.

Levels of the precursor 45S rRNA and the mature 28S, 5.8S, and 18S rRNAs after knockdown of MRPS27 in MDA-MB-231 and BT549 cells determined by qRT-PCR. *$P < 0.05$, **$P < 0.01$, ***$P < 0.001$.

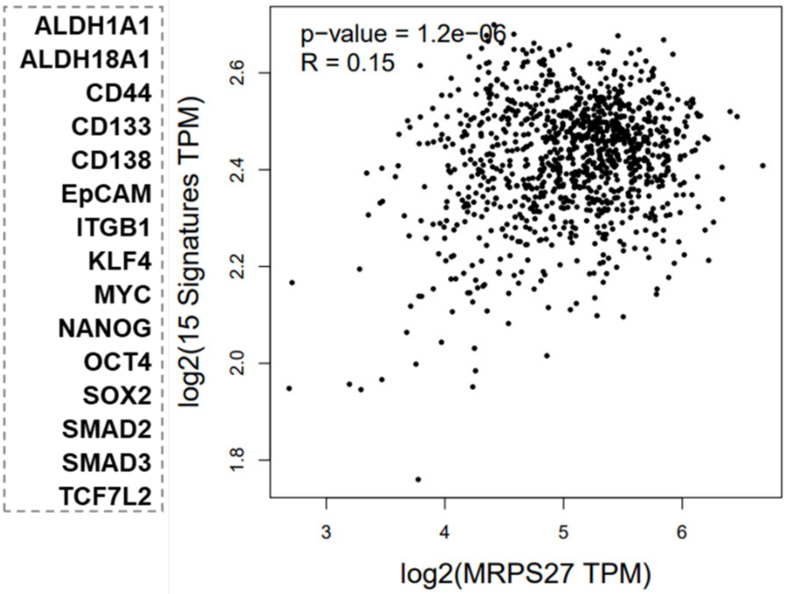

Supplementary Figure S12. MRPS27 is positively correlated with the stemness-related gene signature in breast cancer patients.

The correlation between MRPS27 and the stemness-related gene signature in breast cancer patients analyzed by GEPIA2 database.

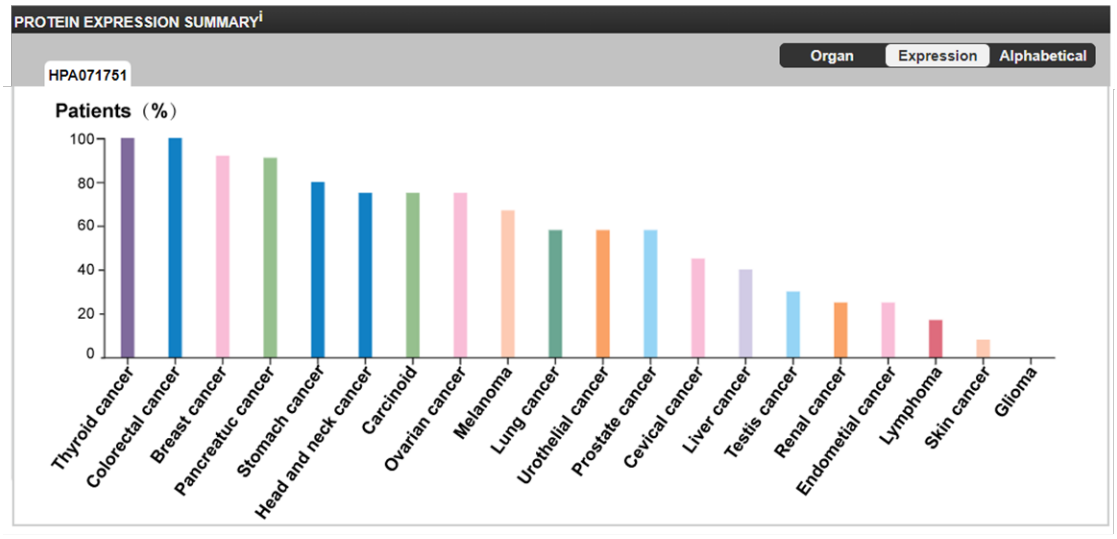

Supplementary Figure S13. MRPS27 is expressed in the majority of breast cancer patients.

The protein levels of MRPS27 in pan-cancer patients based on the HPA database. HPA, Human Protein Atlas.

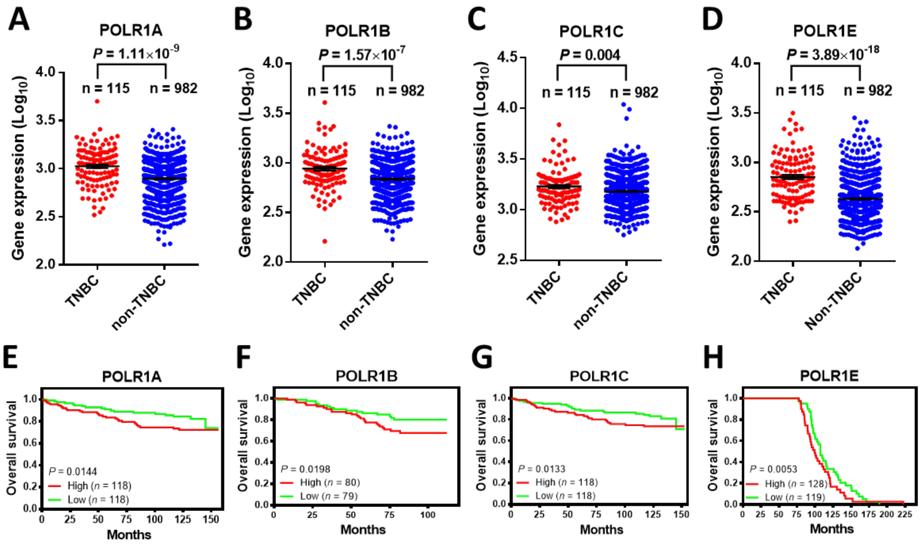

Supplementary Figure S14. RNA polymerase I genes are expressed at higher levels in TNBC and are associated with worse prognosis in breast cancer patients.

(**A-D**) The expression of POLR1A (A), POLR1B (**B**), POLR1C (**C**), and POLR1E (**D**) analyzed between TNBC and non-TNBC clinical samples from The Cancer Genome Atlas (TCGA). (**E-H**) The overall survival in breast cancer patients retrieved from the online database (PROGgeneV2) between high and low expression of POLR1A (**E**, dataset GSE3494_U133B), POLR1B (**F**, dataset GSE1456_U133B), POLR1C (**G**, dataset GSE3494_U133A), and POLR1E (**H**, dataset GSE21653).

Supplementary Table S1. Antibodies used in this study

No.	Name	Source	Identifier	WB Dilution	IF Dilution	IHC Dilution
1	Acetyllysine	PTM BIO	PTM-101	1:1,000		
2	Akt	CST	4685	1:1,000		
3	Akt^{Ser473}	CST	4060	1:1,000		
4	AMPK	CST	2532S	1:1,000		
5	$AMPK^{Thr172}$	CST	2535	1:1,000		
6	CD44	Abcam	ab51037	1:1,000		
7	c-Myc	CST	14962S	1:1,000		
8	Crotonyllysine	PTM BIO	PTM-502	1:1,000		
9	KLF4	ABclonal	A13673	1:2000		
10	Lamin A/C	Proteintech	10298-1-AP	1:5,000		
11	Malonyllysine	PTM BIO	PTM-902	1:1,000		
12	MRPS27	Abcam	Ab153940	1:2000	1:500	1:500
13	mTOR	CST	2983	1:1,000		
14	$mTOR^{Ser2448}$	CST	5536	1:1,000		
15	NOLC1	Sigma	SAB1406798	1:1,000	1:50	
16	NPM	Abcam	ab52664		1:100	
17	Oct4	Abcam	ab109183	1:1,000		
18	p53	CST	9282		1:100	
19	p70S6K	Absin	ab131764	1:1,000		
20	$p70S6K^{Thr389}$	CST	9205	1:1,000		
21	RPL3	ATLAS	HPA003365		1:100	1:400
22	RPS10	Abcam	ab151550		1:300	
23	SOX2	CST	23064	1:1000		
24	Succinyllysine	PTM BIO	PTM-419	1:1,000		
25	c-Myc	ABclonal	A11394			1:200
26	Ki67	CST	9129			1:400
27	β-actin	Bioworld	AP0060	1:5,000		
28	GAPDH	Bioworld	AP0066	1:10,000		
29	Tubulin	Bioworld	AP0064	1:5,000		

WB, western blot; IF, immunofluorescence; IHC, immunohistochemistry

Supplementary Table S2. PCR primers used in this study

No.	Name	Forward	Reverse	Reference or Database
1	45S rRNA	GAACGGTGGTGTGTCGTT	GCGTCTCGTCTCGTCTCACT	Oncogene 31:1254-1263 (2012)
2	28S rRNA	AGAGGTAAACGGGTGGGGTC	GGGGTCGGGAGGAACGG	Oncogene 31:1254-1263 (2012)
3	5.8S rRNA	ACTCGGCTCGTGCGTC	GCGACGCTCAGACAGG	Oncogene 31:1254-1263 (2012)
4	18S rRNA	GATGGTAGTCGCCGTGCC	GCCTGCTGCCTTCCTTGG	Oncogene 31:1254-1263 (2012)
5	MRPS27	ATGGAAACCAGGCTACCTTGA	CCTCGATGTCTAACTGCTCCAC	PrimerBank
6	PUMA	GACGACCTCAACGCACAGTA	AGGAGTCCCATGATGAGATTGT	J Exp Clin Cancer Res 37:97-106 (2018)
7	p21	ATGAAATTCACCCCCTTTCC	CCCTAGGCTGTGCTCACTTC	J Exp Clin Cancer Res 37:97-106 (2018)

Supplementary Table S3. Clinicopathological parameters of 40 cases of breast cancer patients used for iTRAQ proteomics in this study

Patient characteristics	Groups	Patient n (%)
Median age (years, range)	49 (32 – 68)	
Tumor diameter (d, cm), n (%)	d ≤ 2	5 (12.5)
	2 < d ≤5	29 (72.5)
	d > 5	6 (15.0)
Axillary lymph node metastasis, n (%)	N0	13 (32.5)
	N1/2/3	27 (67.5)
Clinical stages, n (%)	Stage II	16 (40.0)
	Stage III	24 (60.0)
Histological type, n (%)	Invasive ductal carcinoma	40 (100.0)
Histological grading, n (%)	Grade I – II	29 (72.5)
	Grade III	11 (27.5)
ER, n (%)	Negative	20 (50.0)
	Positive	20 (50.0)
PR, n (%)	Negative	20 (50.0)
	Positive	20 (50.0)
HER2, n (%)	Negative	20 (50.0)
	Positive	20 (50.0)
Ki67	< 14%	9 (22.5)
	≥ 14%	31 (77.5)
History of menopause, n (%)	Premenopausal	14 (35.0)
	Postmenopausal	26 (65.0)

Supplementary Table S4. shRNAs targeting MRPS27 used in this study

shRNA name	Target sequence
MRPS27 shRNA-1	CCTGCTTTCTTCAGCCTAT
MRPS27 shRNA-2	GCACAAGACAAAGCCCTAT
MRPS27 shRNA-3	GGGCTGTGTACCACAACAT
Control shRNA	TTCTCCGAACGTGTCACGT

Supplementary Table S5. Stem cell frequency in PDX tumors

Group	Cell No.	Sites injected	Positive sites	Estimate	Confidence interval	*P* value
Veh	50000	6	6	1/3348	1/8262-1/1357	-
	5000	6	4			
	500	6	2			
LV-L	50000	6	4	1/20480	1/58095-1/9981	0.001
	5000	6	3			
	500	6	1			
LV-H	50000	6	3	1/61348	1/170343-1/22094	0.0001
	5000	6	1			
	500	6	0			

Veh, vehicle; LV-L, lovastatin-low dose (2 mg/kg); LV-H, lovastatin-high dose (10 mg/kg)

Supplementary Table S6. 10 ribosome biogenesis-related genes in the prognostic model

Num	Gene	UniProt ID	Subcellular location*	Function*
1	MRPS27	Q92552	Mitochondria, nucleolus	Translation regulation, rRNA-binding
2	RRP8	O43159	Nucleoplasm, nucleolus	rRNA processing, transcription regulation
3	TFB1M	Q8WVM0	Mitochondria, cytoplasm	rRNA processing, transcription regulation
4	RPS6KL1	Q9Y6S9	Nucleus, cytoplasm	Ribonucleoprotein, protein phosphorylation
5	DDX11	Q96FC9	Nucleoplasm, nucleolus	rRNA-binding, DNA damage
6	RPS6KA3	P51812	Nucleoplasm, nucleolus	Stress response, cell cycle
7	NR0B1	P51843	Nucleus, cytoplasm	Transcription regulation, RNA polymerase II transcription
8	MDM2	Q00987	Nucleoplasm, cytoplasm	Ubiquitination conjugation, p53 regulation, apoptosis
9	DDX17	Q92841	Nucleoplasm, nucleolus	rRNA processing, mRNA splicing
10	RRP15	Q9Y3B9	Nucleoplasm, nucleolus	rRNA processing, cell cycle, DNA damage

*: Information for subcellular location and protein function is obtained from the Human Protein Atlas database (https://www.proteinatlas.org/).

Supplementary Table S7. The relationship between the expression of MRPS27 and clinicopathologic features in TNBC patients

Clinicopathologic features	MRPS27 Expression		χ^2	*P*
	Low	High		
**Age**			0.3	0.589
< 45	21（55.3%）	17（44.7%）		
⩾ 45	73（50.3%）	72（49.7%）		
**Stage**			4.3	**0.039**
I+II	20（69.0%）	9（31.0%）		
III+IV	74（48.1%）	80（51.9%）		
**T**			1.2	0.273
< 3 cm	17（43.6%）	22（56.4%）		
⩾ 3 cm	77（53.4%）	67（46.6%）		
**N**			4.1	**0.045**
Yes	52（45.6%）	62（54.4%）		
No	42（60.9%）	27（39.1%）		
**M**			0.3	0.593
Yes	2（66.7%）	1（33.3%）		
No	92（51.1%）	88（48.9%）		

T: tumor size, N: lymph node involvement, M: metastases
